# Novel Genetic Variants of Hepatitis B Virus in Fulminant Hepatitis

**DOI:** 10.1155/2017/1231204

**Published:** 2017-12-19

**Authors:** Jack Bee Chook, Yun Fong Ngeow, Kok Keng Tee, Suat Cheng Peh, Rosmawati Mohamed

**Affiliations:** ^1^Sunway Institute for Healthcare Development (SIHD), Sunway University, 47500 Bandar Sunway, Selangor Darul Ehsan, Malaysia; ^2^Department of Pre-Clinical Sciences, Faculty of Medicine and Health Sciences, Universiti Tunku Abdul Rahman, 43000 Selangor, Malaysia; ^3^Department of Medical Microbiology, Faculty of Medicine, University of Malaya, 50603 Kuala Lumpur, Malaysia; ^4^Department of Medicine, Faculty of Medicine, University of Malaya, 50603 Kuala Lumpur, Malaysia

## Abstract

Fulminant hepatitis (FH) is a life-threatening liver disease characterised by intense immune attack and massive liver cell death. The common precore stop codon mutation of hepatitis B virus (HBV), A1896, is frequently associated with FH, but lacks specificity. This study attempts to uncover all possible viral nucleotides that are specifically associated with FH through a compiled sequence analysis of FH and non-FH cases from acute infection. We retrieved 67 FH and 280 acute non-FH cases of hepatitis B from GenBank and applied support vector machine (SVM) model to seek candidate nucleotides highly predictive of FH. Six best candidates with top predictive accuracy, 92.5%, were used to build a SVM model; they are C2129 (85.3%), T720 (83.0%), Y2131 (82.4%), T2013 (82.1%), K2048 (82.1%), and A2512 (82.1%). This model gave a high specificity (99.3%), positive predictive value (95.6%), and negative predictive value (92.1%), but only moderate sensitivity (64.2%). We successfully built a SVM model comprising six variants that are highly predictive and specific for FH: four in the core region and one each in the polymerase and the surface regions. These variants indicate that intracellular virion/core retention could play an important role in the progression to FH.

## 1. Introduction

In hepatitis B virus (HBV) infection, fulminant hepatitis (FH) occurs in less than 1% of adult infections but is associated with nearly 70% mortality [1]. The pathogenesis of FH is largely unclear. This rapidly progressive liver injury is believed to be attributed to a sudden strong cytotoxic T lymphocyte (CTL) response to the presence of HBV, enhanced viral replication, and/or retention of viral capsid in HBV-infected hepatocytes [2, 3]. The result of these events is extensive hepatocyte apoptosis and necrosis leading to an immense loss of liver function that is deadly to HBV-infected individuals.

HBV is classified under the family of Hepadnaviridae. The partially double-stranded DNA genome of the virus contains four overlapping open reading frames (ORF), namely, the polymerase, surface, core, and X genes. While the polymerase and X genes are essential for viral replication, the core gene encodes the core protein (HBcAg) and excretory e antigen (HBeAg), and the surface gene encodes three surface antigens, namely, the large pre-S1, middle pre-S2, and small S proteins [4].

The mutations linked to the development of FH are mainly found in the precore region, the most frequently reported being A1896 [5–7]. A1896 abolishes the expression of HBeAg that is known to induce immune tolerance [8]. It is believed that this removal would lead to the loss of immune tolerance and the consequent enhanced immune attack against hepatocytes. In addition, frame-shift mutations in the precore region have been observed in FH [9]. Germane to this, the predominance of HBV genotypes B and D in FH could be explained by the high occurrence of A1896 in these genotypes as compared to genotypes C and A [10]. Less common mutations, such as G1862T, G1899A, and A2339G, have also been reported [11, 12] but the relevance of these mutations to FH needs further evaluation.

It is currently possible to search for more credible viral genomic variations associated with FH due to increasing numbers of HBV genomes being sequenced worldwide. Through these viral variants, we attempted to provide insights into whether the underlying mechanisms of FH favour host massive immune attack, viral-induced cytopathy due to intracellular virion/capsid accumulation, or both.

## 2. Materials and Methods

### 2.1. Retrieval of HBV Genome Sequences

HBV genomes of FH and non-FH hepatitis B acute infections were retrieved from the National Centre for Biotechnology Information (NCBI) Nucleotide Database on 20 April 2017. The keywords used in advanced search were “Hepatitis B virus”, “complete genome”, and “fulminant” or “acute”. The genome length in the search was restricted to 2,800 to 3,400 nucleotides (nt) as the typical HBV genome size is about 3.2k nt. The initial search produced 98 and 726 hits for FH and non-FH cases, respectively. Acute-on-chronic hepatitis and acute-on-chronic liver failure, which resulted from chronic infection, were excluded from further analysis. After excluding unrelated hits, the final search yielded 67 FH and 280 acute non-FH cases; one case was sub-FH. Acute non-FH cases were used as the control group for sequence comparison in this study. The accession numbers of these HBV genomes are provided in Suppl Table 1. Geographic data of these HBV isolates was also retrieved from GenBank database and published papers wherever available.

### 2.2. Sequence Analysis

HBV genomes were genotyped using the Viral Genotyping Tool available at the NCBI website [13]. Sequence alignment was carried out using MAFFT 6.849 [14]. The alignment was edited using BioEdit [15]. The reference HBV genome used for the numbering of nucleotide positions was D50521.1. The complete HBV genome sequences from FH and non-FH cases were compared and the frequency of each informative candidate nucleotide was determined.

### 2.3. Phylogenetic Analysis

A neighbour-joining (NJ) tree was reconstructed for the complete genomes of HBV in the phylogenetic analysis, using MEGA version 7.0.18 software, with a Kimura-2 parameter nucleotide substitution model, uniform rate among sites, and complete deletion for gaps [16–18]. The reliability of the branching orders was evaluated by a bootstrap analysis of 1,000 replicates.

### 2.4. Statistical Analysis and Interpretation of Results

Statistical analyses were performed using the Statistical Program for Social Sciences (SPSS 17.0 for windows, SPSS, Inc., Chicago, IL). Fisher's exact test was applied to examine the association between nucleotide type and disease state. A *P* value of <0.05 was taken as significant.

### 2.5. Support Vector Machine Modelling

To avoid false positive discovery, observations were randomised 20 times to benchmark the maximum random guess accuracy; in this case, it was found to be 81.8% (284/347). Fifty-five informative candidate nucleotides that passed the maximum random guess accuracy were identified based on the MAFFT-aligned sequence data. These 55 nucleotides were sorted by accuracy in a descending order before proceeding to feature selection processes. Single round of brute force selection of 6 candidate nucleotides at a time using the 55 nucleotides is not possible as it requires high computational resource. Two rounds of brute force selection procedures were implemented with support vector machine (SVM) dot kernel algorithm in RapidMiner 7.2.001 [19, 20]. The first round generated a list consisting of three candidate nucleotides in combination that gave the highest accuracy. The top 25 most informative candidate nucleotides were determined in this round. The second round of brute force selection was then applied to further reduce the number of the nucleotides to a maximum of six, in order to minimise the risk of overfitting the SVM model.

The SVM model was then used to assess the predictive values of the candidate nucleotides thus selected. For each feature, the most frequent nucleotide type across both classes was coded as 1 and others were coded as 0. To reduce biases due to uneven sampling size, the predictive accuracy of the candidate nucleotides was assessed using a balanced training dataset consisting of 67 FH cases and 67 randomly chosen cases from the control group. This was to benchmark the random guess accuracy at 50%. The random sampling for cases from control group was repeated 1,000 times. A 5-fold cross-validation was carried out for the 1,000 balanced datasets.

Predictive values were expressed in terms of overall or average accuracy, sensitivity, specificity, positive predictive value (PPV), and negative predictive value (NPV). “Overall” referred to all cases and controls, whereas “average” was used for the calculation of predictive values in 5-fold cross-validation using the balanced sampling method. Accuracy was defined as the sum of correct guesses of FH and acute non-FH cases over the total number of both cases. Sensitivity was calculated by dividing the sum of correctly identified FH cases with a marker over all FH cases, whereas specificity was obtained by dividing the sum of correctly identified control cases without the marker over all control cases. The PPV was calculated by dividing the sum of correctly identified FH cases over all positive predictions, and the NPV was calculated by dividing correctly identified control cases over all negative predictions.

## 3. Results

### 3.1. Geographical Distribution, Genotype, and Phylogenetic Analysis

Of 67 cases in FH, 44 were from Asian countries (mainly China and Japan; 65.7%), 23.9% from the West (Europe and the USA), 6 from Africa (9.0%), and 1 from the Middle East (1.5%), whereas, in 280 acute non-FH cases, the corresponding percentages were 169 from Asia (60.4%), 50 from the West (17.9%), 5 from Africa (1.8%), and 56 from South America (20.0%). The geographical distribution of FH and non-FH groups seems to be similar. Most of the HBV are of genotypes A–D (Table 1). Genotypes B and D are predominant in FH cases as compared with acute non-FH cases (*P* < 0.001). Genotypes F, G, H, and I were only seen in acute non-FH cases. Phylogenetic analysis of HBV genotypes A–D (Suppl Figure 1) showed that FH cases and controls did not form distinct clusters. However, some cases clustered together due to either similar geographical location (such as Belgium cases in acute hepatitis) or distinct genotype (such as genotype F cases in acute hepatitis) in single disease outcome study.

### 3.2. Viral Candidate Nucleotides Potentially Predictive of FH

A total of 55 informative candidate nucleotides that passed the maximum random guess accuracy were identified (Suppl Table 2). These nucleotides were sorted in the descending order based on accuracy values. The top 25 were chosen in the first round of brute force selection in a list of combination of three nucleotides; they were A1410, A2345, A2512, B3106, C123, C132, C280, C774, C2129, D2303, G1981, G2003, G2173, G2430, G2431, K2048, T343, T720, T2013, T2755, T2979, W1677, Y2092, and Y2131. These 25 nucleotides proceeded to the second round of brute force selection. A list consisting of 5-6 candidate nucleotides in combinations was generated using SVM modelling (Suppl Table 3). The list with the highest accuracy, 92.51%, was selected; they are T720, T2013, K2048, C2129, Y2131, and A2512 (Table 2). Two variants coexist in some FH cases (Suppl Table 4). A 5-fold cross-validation method repeated 1,000 times was applied to evaluate the predictability of the candidate nucleotides in the list, and balanced sampling size (*N* = 67) was adopted to reduce the biases in data analysis. Average predictive accuracy was calculated to be 81.1%  ± 0.70%, sensitivity 63.7%  ± 1.41%, specificity 98.5%  ± 0.00%, PPV 97.7%  ± 0.05%, and NPV 73.1%  ± 0.74%. Overall predictive accuracy was 92.5%, sensitivity 64.2%, specificity 99.3%, PPV 95.6%, and NPV 92.1%.

## 4. Discussion

FH is a dreaded complication of HBV infection. The identification of FH-associated HBV mutations could provide a more precise insight into the disease pathogenesis of FH. We built a SVM model that is highly predictive and specific for the development of FH. It consists of six novel HBV nucleotide variants, four of which are in the core gene. Here, we attempt to explain the rationales of these nucleotide markers in the pathogenesis of FH.

The core protein, also known as HBcAg, has 183 residues, of which 149 at the N-terminus belong to the assembly domain, and 34 at the C-terminus are the RNA-binding domain [21–23]. All four of the core variants we identified, T2013, K2048, C2129, and Y2131, corresponding to residues 38, 50, 77, and 77, respectively, fall within the assembly domain, which forms the shell of viral capsid. Mutations in this domain may cause inefficient capsid assembly and subsequently retention of capsid within hepatocytes that could cause liver injury [24]. In a study on HBV-infected human liver biopsies, the cytoplasmic localisation of HBcAg was related to more hepatic injury [25]. Immunohistochemical examination of liver tissue samples showed stronger staining of HBcAg in the nucleus of tissues from FH patients than in tissues from acute hepatitis patients [2]. These two studies indicate that excessive intracellular accumulation of virion/HBcAg is cytopathic to hepatocytes.

At residue 77 of HBcAg, there is a G-C (Glu to Gln) switch in nucleotide 2129 (Glu77Gln; codons GAA to CAA) that is accompanied by a change in charge from acidic to polar uncharged (but no change in secondary structure), as well as an A-Y (Glu to Asp) change in nucleotide 2131 (GAA to GAY), which is accompanied by a change in secondary structure from alpha-helix to turn-and-loop (but no change in charge). These G2129C and A2131Y changes could be important for the intracellular retention of the HBV virion. Supporting evidence for this hypothesis comes from the observations of other workers that concomitant changes of Glu77Gln with Pro79Gln, Ala80Pro, and Ser181Pro in the core protein appear to promote nuclear retention of HBcAg [24] and that Glu77Ala in silico destabilises the core residue-surface antigen interface which is essential for the secretion of virions after assembly [26].

In the A2013T variant, there is a switch from Tyr to Phe (TAT to TTT). Tyr can be phosphorylated and glycosylated, but not Phe. In general, the phosphorylation of amino acids plays a role in signalling, whereas glycosylation affects protein secretion, structural formation, and antigenicity. Although there is no change in structure and charge in A2013T, an alteration in the posttranslational modification capability can potentially affect the assembly efficiency of the viral capsid. Failure in the HBcAg assembly might then cause excessive accumulation of viral capsids within hepatocytes.

In C2048K, corresponding to residue 38, there is a switch from Pro to Ala/Tyr (CCT to GCT/TAT) at residue 50, along with a change in charge from polar to nonpolar. Such an amino acid alteration might hinder the formation of the turn-and-loop structure in HBcAg. As C2048K has not, so far, been implicated in disease pathogenesis, the impact of this variant on virion/core protein retention warrants further investigation. In addition, C2048K is located within T helper CD4+ epitopes of HBcAg [27] and, hence, is expected to affect host immune response to HBV. However, there is no direct evidence that structural change in the residue 50 in the Th CD4+ epitope could invoke a strong immune attack on hepatocytes.

The C720T variant is corresponding to residue 189 of HBsAg. This substitution of Thr (ACT) with Ile (ATT) is not associated with structural change but a shift from polar to nonpolar charge state. The residues 96–122 and residues 169–195 of HBsAg participate in the capsid interaction with core protein in residues 67–96 in order to promote the formation of an infectious virus [26]. The absence of such an interaction may cause intracellular retention of virions, which subsequently induce cytopathic effects in hepatocytes. The hepatitis B small surface antigen (HBsAg) is believed to have at least 3 transmembrane domains (TM1, TM2, and TM3) [28]. The C720T variant falls within the TM3 domain (containing residues 161–226). Nonsynonymous amino acid mutations, such as S167L and Q181STOP, in the TM3 domain, reduce HBsAg reactivity in occult HBV infections [29, 30]. However, whether the C720T variant (T189I) possesses immune-escape capability that allows excessive accumulation of virions within hepatocyte requires further investigation.

Terminal (TP) is one of the protein domains in the polymerase enzyme. It acts as a protein primer for viral DNA synthesis. TP-epsilon RNA binding is required for the packaging of both polymerase and pregenomic RNAs (pgRNA) into nucleocapsids [31]. TP residues 67–80 are critical for such binding [32]. B2512A corresponds to residue 69 of TP. The residue falls within the TP priming loop region. In acute non-FH cases, it seems to be heterogeneous. B2512A converts Ile (ATC), Val (GTC), Cys (TGC), or Thr (ACT) to Asn (AAY). The former three amino acid residues incline to form a beta-sheet structure, whereas the latter one hinders the formation of the beta-sheet structure. Such structural hindrance could inhibit the TP-RNA-binding function. Consequently, this would cause accumulation of viral capsids and pregenomic RNAs (pgRNAs) in hepatocytes.

Surprisingly, A1896, the most commonly cited marker for FH, is not among the 6 variants selected for building the SVM model. This could be due to its low predictive specificity. The link between A1896 and FH has been demonstrated in Japanese studies [33, 34], but not in studies from other countries like United States and Germany [35]. We also did not find HBV genotyping to be useful for the identification of FH cases as genotypes A–D are associated with both FH and acute non-FH. On the other hand, we did not find genotypes F–I in any of the FH cases in our series. Hence, these genotypes could be investigated further for their potential significance as biomarkers for non-FH.

In conclusion, we identified six HBV variants (4 in the core, 1 in the polymerase-TP, and 1 in the surface/polymerase-RT region) that are highly predictive and specific for the identification of patients at risk for FH. These viral variants could play an important role in the disease progression to FH by causing excessive accumulation of viral capsids in hepatocytes. Hence, our findings favour the hypothesis of viral-induced cell death over hepatic immune attack, being an important factor in the pathogenesis of FH.

## Figures and Tables

**Table 1:** Genotype distribution of fulminant and acute nonfulminant hepatitis B viral genomes retrieved from NCBI.

Genotype	Fulminant, *N* = 67 (%)	Acute, *N* = 280 (%)
A	8 (11.9)	65 (23.2)
B	22 (32.8)	31 (11.1)
C	22 (32.8)	112 (40.0)
D	15 (22.4)	26 (9.3)
F	0 (0.0)	40 (14.3)
G	0 (0.0)	1 (0.4)
H	0 (0.0)	4 (1.4)
I	0 (0.0)	1 (0.4)

NCBI: National Center for Biotechnology Information.

**Table 2:** The best combination of six candidate nucleotides of HBV associated with fulminant hepatitis was chosen from top 55 candidate nucleotides using brute force selection method implemented with SVM dot kernel algorithm.

Nucleotide	Gene/regulatory element	Fulminant, *n* = 67 (% sensitivity)	Acute, *n* = 280 (% specificity)	% accuracy^a^	*P* value^b^
C2129	Core	17 (25.4)	1 (99.6)	85.3	1.897*E* − 12
T720	Surface/polymerase-RT	9 (13.4)	1 (99.6)	83.0	2.000*E* − 06
Y2131	Core	6 (9.0)	0 (100.0)	82.4	4.300*E* − 05
T2013	Core	5 (7.5)	0 (100.0)	82.1	2.371*E* − 04
K2048	Core	5 (7.5)	0 (100.0)	82.1	2.371*E* − 04
A2512	Polymerase-TP	5 (7.5)	0 (100.0)	82.1	2.371*E* − 04

HBV, hepatitis B virus; SVM, support vector machine; RT, reverse transcriptase; TP, terminal protein; ^a^% accuracy = (67 ×  % sensitivity + 280 ×  % specificity)/(67 + 280); ^b^Fisher's exact test; *P* value of 1.897*E* − 12 is equivalent to 1.897 × 10^−12^.

## Supplementary Materials

Supplementary 1 Suppl Figure  1: the evolutionary history was inferred using the neighbour-joining method. The percentage of replicate trees in which the associated taxa clustered together in the bootstrap test (1000 replicates) are shown next to the branches. The tree is drawn to scale, with branch lengths in the same units as those of the evolutionary distances used to infer the phylogenetic tree. The evolutionary distances were computed using the Kimura 2-parameter method and are in the units of the number of base substitutions per site. The analysis involved 347 nucleotide sequences. All positions containing gaps and missing data were eliminated. There were a total of 2,362 positions in the final dataset. Evolutionary analyses were conducted in MEGA7.

Supplementary 2 Suppl Table  1: genotype and geographical distribution of fulminant and acute hepatitis B.

Supplementary 3 Suppl Table  2: top 55 candidate nucleotides associated with fulminant hepatitis B.

Supplementary 4 Suppl Table  3: list of top 25 most informative nucleotide variants associated with fulminant hepatitis B in combinations generated using SVM modelling with brute force selection.

Supplementary 5 Suppl Table  4: frequency of top 6 nucleotide variants in HBV isolates of fulminant and acute hepatitis.
