# Supplementary material for: Novel Genetic Variants of Hepatitis B Virus in Fulminant Hepatitis
Source: J Pathog. 2017 Dec 19;2017:1231204. doi: 10.1155/2017/1231204 (PMC5749291; doi:10.1155/2017/1231204)
Supplement: Supplementary 1 — Suppl Figure 1: the evolutionary history was inferred using the neighbour-joining method. The percentage of replicate trees in which the associated taxa clustered together in the bootstrap test (1000 replicates) are shown next to the branches. The tree is drawn to scale, with branch lengths in the same units as those of the evolutionary distances used to infer the phylogenetic tree. The evolutionary distances were computed using the Kimura 2-parameter method and are in the units of the number of base substitutions per site. The analysis involved 347 nucleotide sequences. All positions containing gaps and missing data were eliminated. There were a total of 2,362 positions in the final dataset. Evolutionary analyses were conducted in MEGA7. [file 1231204.f1.pdf]

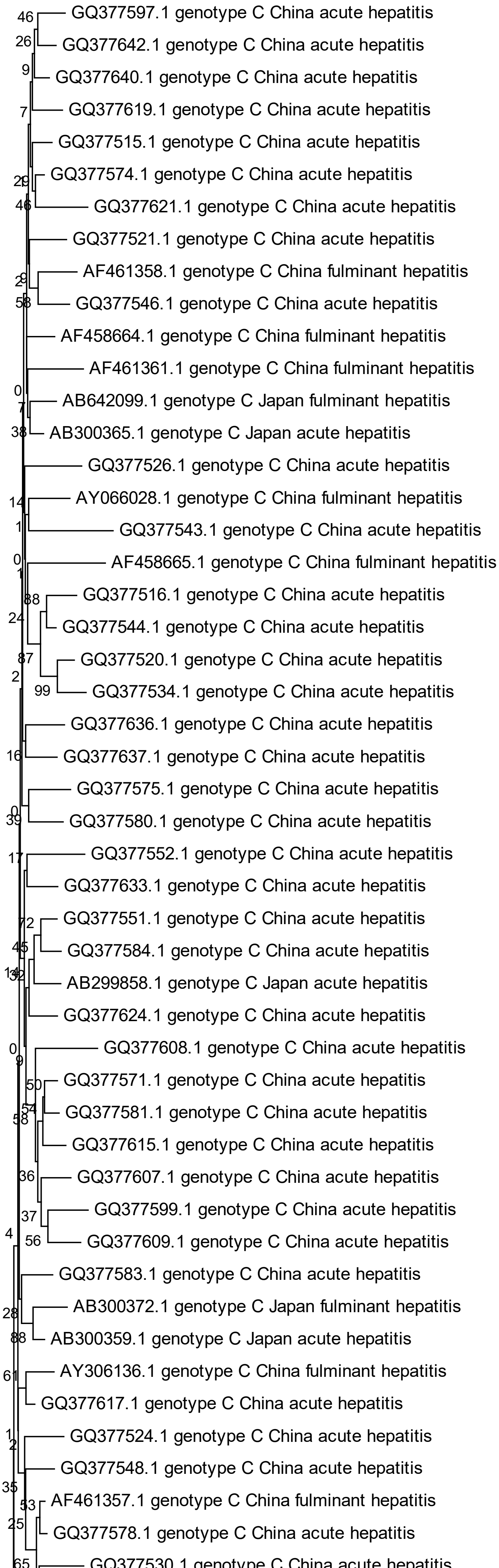
GQ377597.1 genotype C China acute hepatitis
GQ377642.1 genotype C China acute hepatitis
GQ377640.1 genotype C China acute hepatitis
GQ377619.1 genotype C China acute hepatitis
GQ377515.1 genotype C China acute hepatitis
GQ377574.1 genotype C China acute hepatitis
GQ377621.1 genotype C China acute hepatitis
GQ377521.1 genotype C China acute hepatitis
AF461358.1 genotype C China fulminant hepatitis
GQ377546.1 genotype C China acute hepatitis
AF458664.1 genotype C China fulminant hepatitis
AF461361.1 genotype C China fulminant hepatitis
AB642099.1 genotype C Japan fulminant hepatitis
AB300365.1 genotype C Japan acute hepatitis
GQ377526.1 genotype C China acute hepatitis
AY066028.1 genotype C China fulminant hepatitis
GQ377543.1 genotype C China acute hepatitis
AF458665.1 genotype C China fulminant hepatitis
GQ377516.1 genotype C China acute hepatitis
GQ377544.1 genotype C China acute hepatitis
GQ377520.1 genotype C China acute hepatitis
GQ377534.1 genotype C China acute hepatitis
GQ377636.1 genotype C China acute hepatitis
GQ377637.1 genotype C China acute hepatitis
GQ377575.1 genotype C China acute hepatitis
GQ377580.1 genotype C China acute hepatitis
GQ377552.1 genotype C China acute hepatitis
GQ377633.1 genotype C China acute hepatitis
GQ377551.1 genotype C China acute hepatitis
GQ377584.1 genotype C China acute hepatitis
AB299858.1 genotype C Japan acute hepatitis
GQ377624.1 genotype C China acute hepatitis
GQ377608.1 genotype C China acute hepatitis
GQ377571.1 genotype C China acute hepatitis
GQ377581.1 genotype C China acute hepatitis
GQ377615.1 genotype C China acute hepatitis
GQ377607.1 genotype C China acute hepatitis
GQ377599.1 genotype C China acute hepatitis
GQ377609.1 genotype C China acute hepatitis
GQ377583.1 genotype C China acute hepatitis
AB300372.1 genotype C Japan fulminant hepatitis
AB300359.1 genotype C Japan acute hepatitis
AY306136.1 genotype C China fulminant hepatitis
GQ377617.1 genotype C China acute hepatitis
GQ377524.1 genotype C China acute hepatitis
GQ377548.1 genotype C China acute hepatitis
AF461357.1 genotype C China fulminant hepatitis
GQ377578.1 genotype C China acute hepatitis

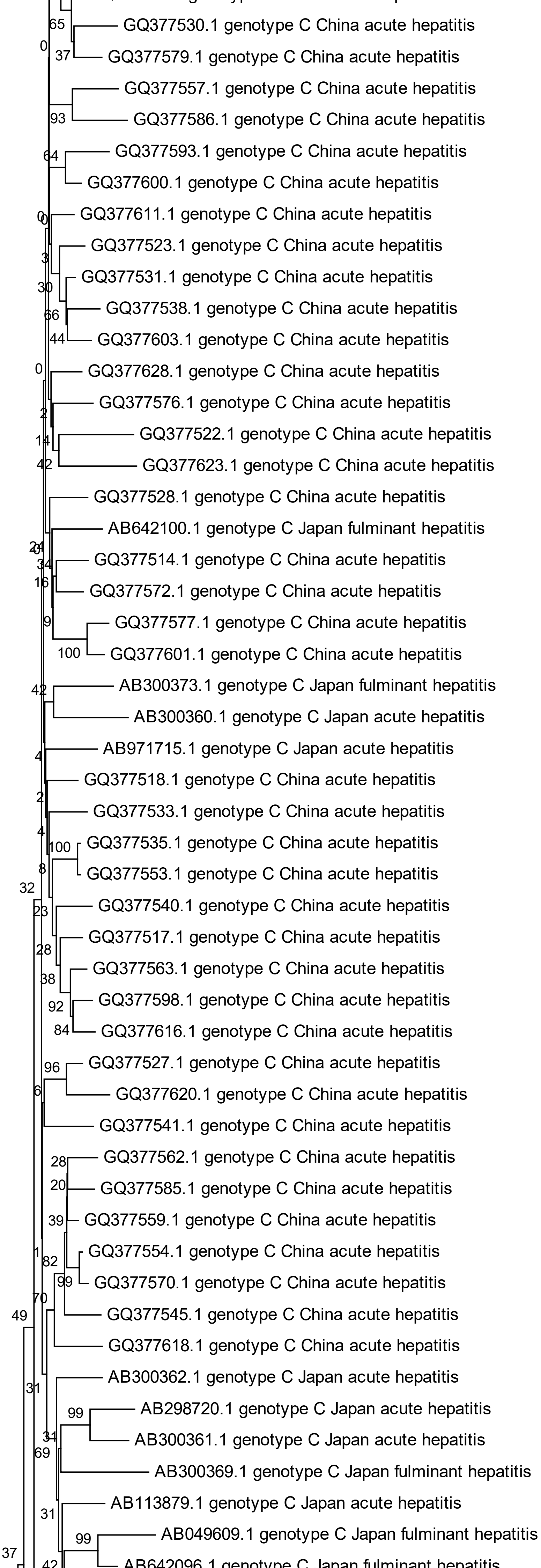
GQ377530.1 genotype C China acute hepatitis
GQ377579.1 genotype C China acute hepatitis
GQ377557.1 genotype C China acute hepatitis
GQ377586.1 genotype C China acute hepatitis
GQ377593.1 genotype C China acute hepatitis
GQ377600.1 genotype C China acute hepatitis
GQ377611.1 genotype C China acute hepatitis
GQ377523.1 genotype C China acute hepatitis
GQ377531.1 genotype C China acute hepatitis
GQ377538.1 genotype C China acute hepatitis
GQ377603.1 genotype C China acute hepatitis
GQ377628.1 genotype C China acute hepatitis
GQ377576.1 genotype C China acute hepatitis
GQ377522.1 genotype C China acute hepatitis
GQ377623.1 genotype C China acute hepatitis
GQ377528.1 genotype C China acute hepatitis
AB642100.1 genotype C Japan fulminant hepatitis
GQ377514.1 genotype C China acute hepatitis
GQ377572.1 genotype C China acute hepatitis
GQ377577.1 genotype C China acute hepatitis
GQ377601.1 genotype C China acute hepatitis
AB300373.1 genotype C Japan fulminant hepatitis
AB300360.1 genotype C Japan acute hepatitis
AB971715.1 genotype C Japan acute hepatitis
GQ377518.1 genotype C China acute hepatitis
GQ377533.1 genotype C China acute hepatitis
GQ377535.1 genotype C China acute hepatitis
GQ377553.1 genotype C China acute hepatitis
GQ377540.1 genotype C China acute hepatitis
GQ377517.1 genotype C China acute hepatitis
GQ377563.1 genotype C China acute hepatitis
GQ377598.1 genotype C China acute hepatitis
GQ377616.1 genotype C China acute hepatitis
GQ377527.1 genotype C China acute hepatitis
GQ377620.1 genotype C China acute hepatitis
GQ377541.1 genotype C China acute hepatitis
GQ377562.1 genotype C China acute hepatitis
GQ377585.1 genotype C China acute hepatitis
GQ377559.1 genotype C China acute hepatitis
GQ377554.1 genotype C China acute hepatitis
GQ377570.1 genotype C China acute hepatitis
GQ377545.1 genotype C China acute hepatitis
GQ377618.1 genotype C China acute hepatitis
AB300362.1 genotype C Japan acute hepatitis
AB298720.1 genotype C Japan acute hepatitis
AB300361.1 genotype C Japan acute hepatitis
AB300369.1 genotype C Japan fulminant hepatitis
AB113879.1 genotype C Japan acute hepatitis
AB049609.1 genotype C Japan fulminant hepatitis
AB642096.1 genotype C Japan fulminant hepatitis

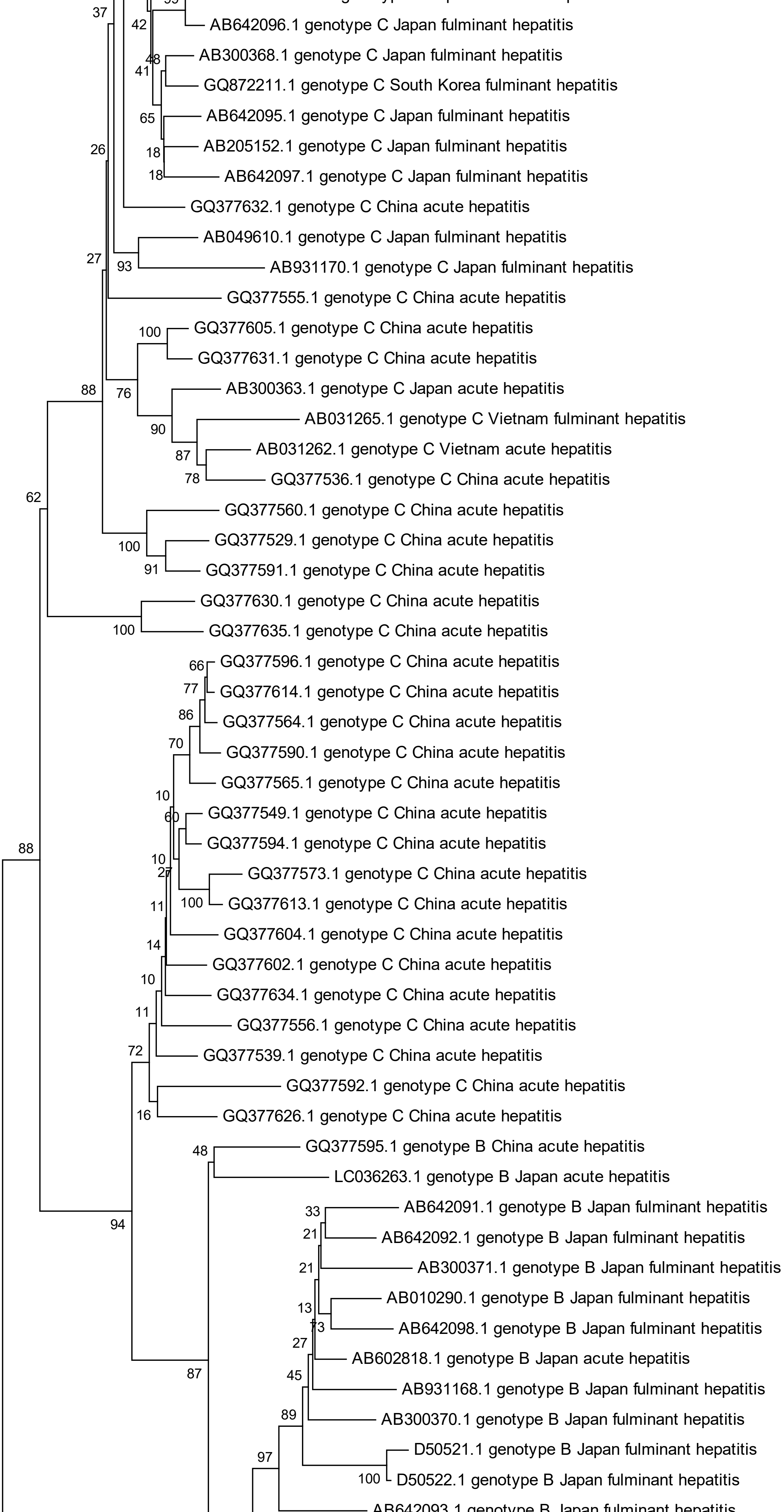
AB642096.1 genotype C Japan fulminant hepatitis
AB300368.1 genotype C Japan fulminant hepatitis
GQ872211.1 genotype C South Korea fulminant hepatitis
AB642095.1 genotype C Japan fulminant hepatitis
AB205152.1 genotype C Japan fulminant hepatitis
AB642097.1 genotype C Japan fulminant hepatitis
GQ377632.1 genotype C China acute hepatitis
AB049610.1 genotype C Japan fulminant hepatitis
AB931170.1 genotype C Japan fulminant hepatitis
GQ377555.1 genotype C China acute hepatitis
GQ377605.1 genotype C China acute hepatitis
GQ377631.1 genotype C China acute hepatitis
AB300363.1 genotype C Japan acute hepatitis
AB031265.1 genotype C Vietnam fulminant hepatitis
AB031262.1 genotype C Vietnam acute hepatitis
GQ377536.1 genotype C China acute hepatitis
GQ377560.1 genotype C China acute hepatitis
GQ377529.1 genotype C China acute hepatitis
GQ377591.1 genotype C China acute hepatitis
GQ377630.1 genotype C China acute hepatitis
GQ377635.1 genotype C China acute hepatitis
GQ377596.1 genotype C China acute hepatitis
GQ377614.1 genotype C China acute hepatitis
GQ377564.1 genotype C China acute hepatitis
GQ377590.1 genotype C China acute hepatitis
GQ377565.1 genotype C China acute hepatitis
GQ377549.1 genotype C China acute hepatitis
GQ377594.1 genotype C China acute hepatitis
GQ377573.1 genotype C China acute hepatitis
GQ377613.1 genotype C China acute hepatitis
GQ377604.1 genotype C China acute hepatitis
GQ377602.1 genotype C China acute hepatitis
GQ377634.1 genotype C China acute hepatitis
GQ377556.1 genotype C China acute hepatitis
GQ377539.1 genotype C China acute hepatitis
GQ377592.1 genotype C China acute hepatitis
GQ377626.1 genotype C China acute hepatitis
GQ377595.1 genotype B China acute hepatitis
LC036263.1 genotype B Japan acute hepatitis
AB642091.1 genotype B Japan fulminant hepatitis
AB642092.1 genotype B Japan fulminant hepatitis
AB300371.1 genotype B Japan fulminant hepatitis
AB010290.1 genotype B Japan fulminant hepatitis
AB642098.1 genotype B Japan fulminant hepatitis
AB602818.1 genotype B Japan acute hepatitis
AB931168.1 genotype B Japan fulminant hepatitis
AB300370.1 genotype B Japan fulminant hepatitis
D50521.1 genotype B Japan fulminant hepatitis
D50522.1 genotype B Japan fulminant hepatitis

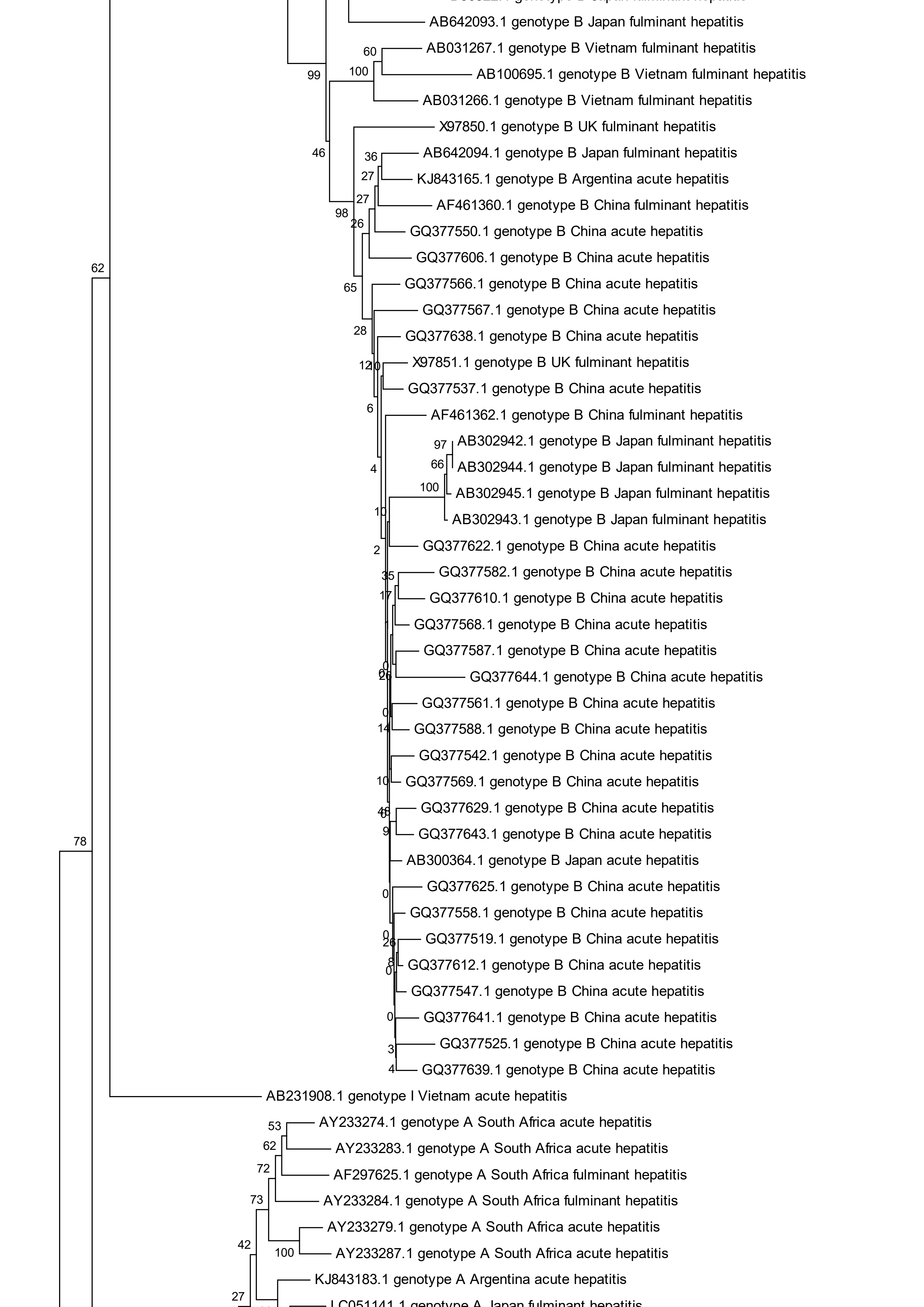
AB642093.1 genotype B Japan fulminant hepatitis
AB031267.1 genotype B Vietnam fulminant hepatitis
AB100695.1 genotype B Vietnam fulminant hepatitis
AB031266.1 genotype B Vietnam fulminant hepatitis
X97850.1 genotype B UK fulminant hepatitis
AB642094.1 genotype B Japan fulminant hepatitis
KJ843165.1 genotype B Argentina acute hepatitis
AF461360.1 genotype B China fulminant hepatitis
GQ377550.1 genotype B China acute hepatitis
GQ377606.1 genotype B China acute hepatitis
GQ377566.1 genotype B China acute hepatitis
GQ377567.1 genotype B China acute hepatitis
GQ377638.1 genotype B China acute hepatitis
X97851.1 genotype B UK fulminant hepatitis
GQ377537.1 genotype B China acute hepatitis
AF461362.1 genotype B China fulminant hepatitis
AB302942.1 genotype B Japan fulminant hepatitis
AB302944.1 genotype B Japan fulminant hepatitis
AB302945.1 genotype B Japan fulminant hepatitis
AB302943.1 genotype B Japan fulminant hepatitis
GQ377622.1 genotype B China acute hepatitis
GQ377582.1 genotype B China acute hepatitis
GQ377610.1 genotype B China acute hepatitis
GQ377568.1 genotype B China acute hepatitis
GQ377587.1 genotype B China acute hepatitis
GQ377644.1 genotype B China acute hepatitis
GQ377561.1 genotype B China acute hepatitis
GQ377588.1 genotype B China acute hepatitis
GQ377542.1 genotype B China acute hepatitis
GQ377569.1 genotype B China acute hepatitis
GQ377629.1 genotype B China acute hepatitis
GQ377643.1 genotype B China acute hepatitis
AB300364.1 genotype B Japan acute hepatitis
GQ377625.1 genotype B China acute hepatitis
GQ377558.1 genotype B China acute hepatitis
GQ377519.1 genotype B China acute hepatitis
GQ377612.1 genotype B China acute hepatitis
GQ377547.1 genotype B China acute hepatitis
GQ377641.1 genotype B China acute hepatitis
GQ377525.1 genotype B China acute hepatitis
GQ377639.1 genotype B China acute hepatitis
AB231908.1 genotype I Vietnam acute hepatitis
AY233274.1 genotype A South Africa acute hepatitis
AY233283.1 genotype A South Africa acute hepatitis
AF297625.1 genotype A South Africa fulminant hepatitis
AY233284.1 genotype A South Africa fulminant hepatitis
AY233279.1 genotype A South Africa acute hepatitis
AY233287.1 genotype A South Africa acute hepatitis
KJ843183.1 genotype A Argentina acute hepatitis
LC051141.1 genotype A Japan fulminant hepatitis

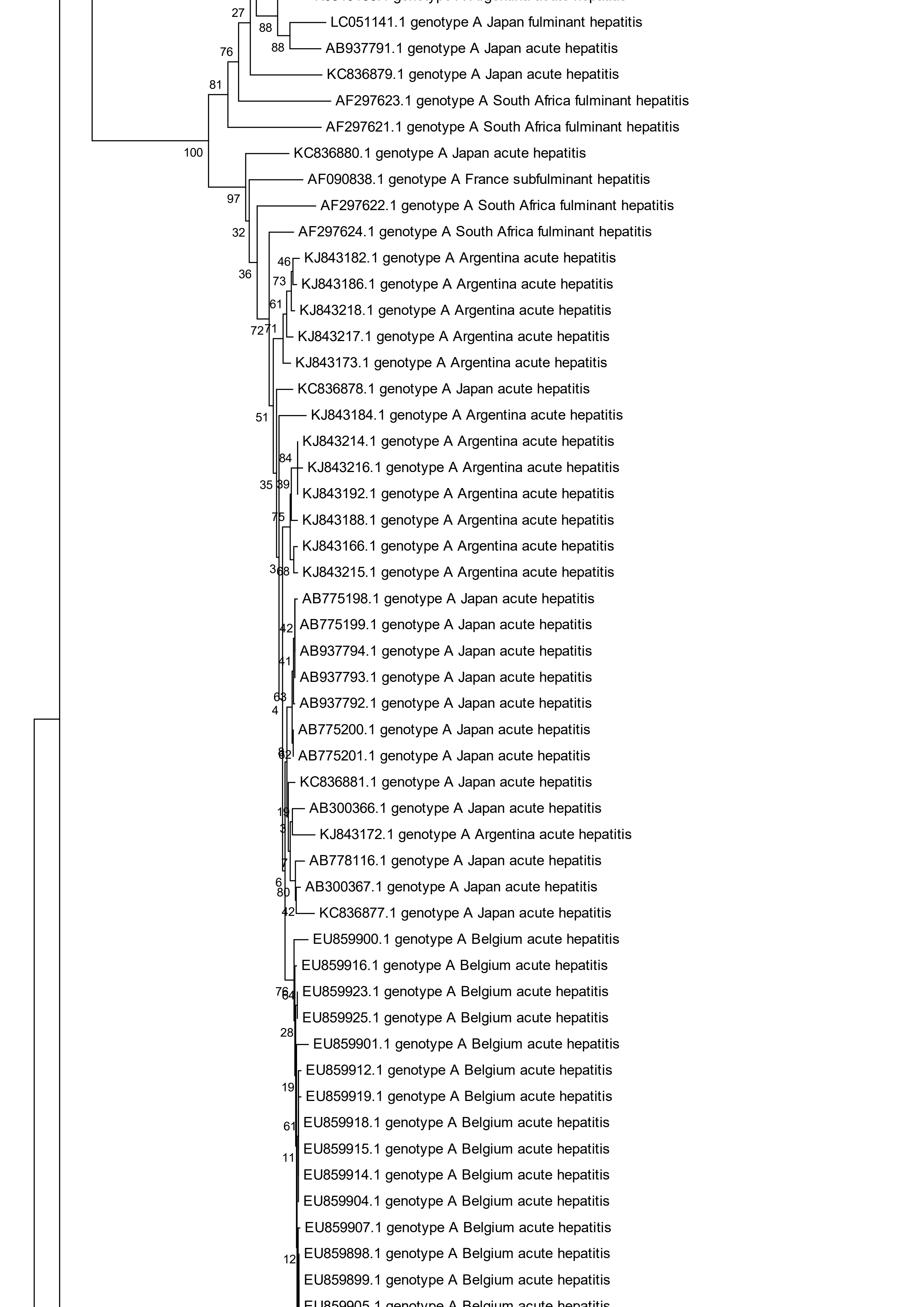
LC051141.1 genotype A Japan fulminant hepatitis
AB937791.1 genotype A Japan acute hepatitis
KC836879.1 genotype A Japan acute hepatitis
AF297623.1 genotype A South Africa fulminant hepatitis
AF297621.1 genotype A South Africa fulminant hepatitis
KC836880.1 genotype A Japan acute hepatitis
AF090838.1 genotype A France subfulminant hepatitis
AF297622.1 genotype A South Africa fulminant hepatitis
AF297624.1 genotype A South Africa fulminant hepatitis
KJ843182.1 genotype A Argentina acute hepatitis
KJ843186.1 genotype A Argentina acute hepatitis
KJ843218.1 genotype A Argentina acute hepatitis
KJ843217.1 genotype A Argentina acute hepatitis
KJ843173.1 genotype A Argentina acute hepatitis
KC836878.1 genotype A Japan acute hepatitis
KJ843184.1 genotype A Argentina acute hepatitis
KJ843214.1 genotype A Argentina acute hepatitis
KJ843216.1 genotype A Argentina acute hepatitis
KJ843192.1 genotype A Argentina acute hepatitis
KJ843188.1 genotype A Argentina acute hepatitis
KJ843166.1 genotype A Argentina acute hepatitis
KJ843215.1 genotype A Argentina acute hepatitis
AB775198.1 genotype A Japan acute hepatitis
AB775199.1 genotype A Japan acute hepatitis
AB937794.1 genotype A Japan acute hepatitis
AB937793.1 genotype A Japan acute hepatitis
AB937792.1 genotype A Japan acute hepatitis
AB775200.1 genotype A Japan acute hepatitis
AB775201.1 genotype A Japan acute hepatitis
KC836881.1 genotype A Japan acute hepatitis
AB300366.1 genotype A Japan acute hepatitis
KJ843172.1 genotype A Argentina acute hepatitis
AB778116.1 genotype A Japan acute hepatitis
AB300367.1 genotype A Japan acute hepatitis
KC836877.1 genotype A Japan acute hepatitis
EU859900.1 genotype A Belgium acute hepatitis
EU859916.1 genotype A Belgium acute hepatitis
EU859923.1 genotype A Belgium acute hepatitis
EU859925.1 genotype A Belgium acute hepatitis
EU859901.1 genotype A Belgium acute hepatitis
EU859912.1 genotype A Belgium acute hepatitis
EU859919.1 genotype A Belgium acute hepatitis
EU859918.1 genotype A Belgium acute hepatitis
EU859915.1 genotype A Belgium acute hepatitis
EU859914.1 genotype A Belgium acute hepatitis
EU859904.1 genotype A Belgium acute hepatitis
EU859907.1 genotype A Belgium acute hepatitis
EU859898.1 genotype A Belgium acute hepatitis
EU859899.1 genotype A Belgium acute hepatitis

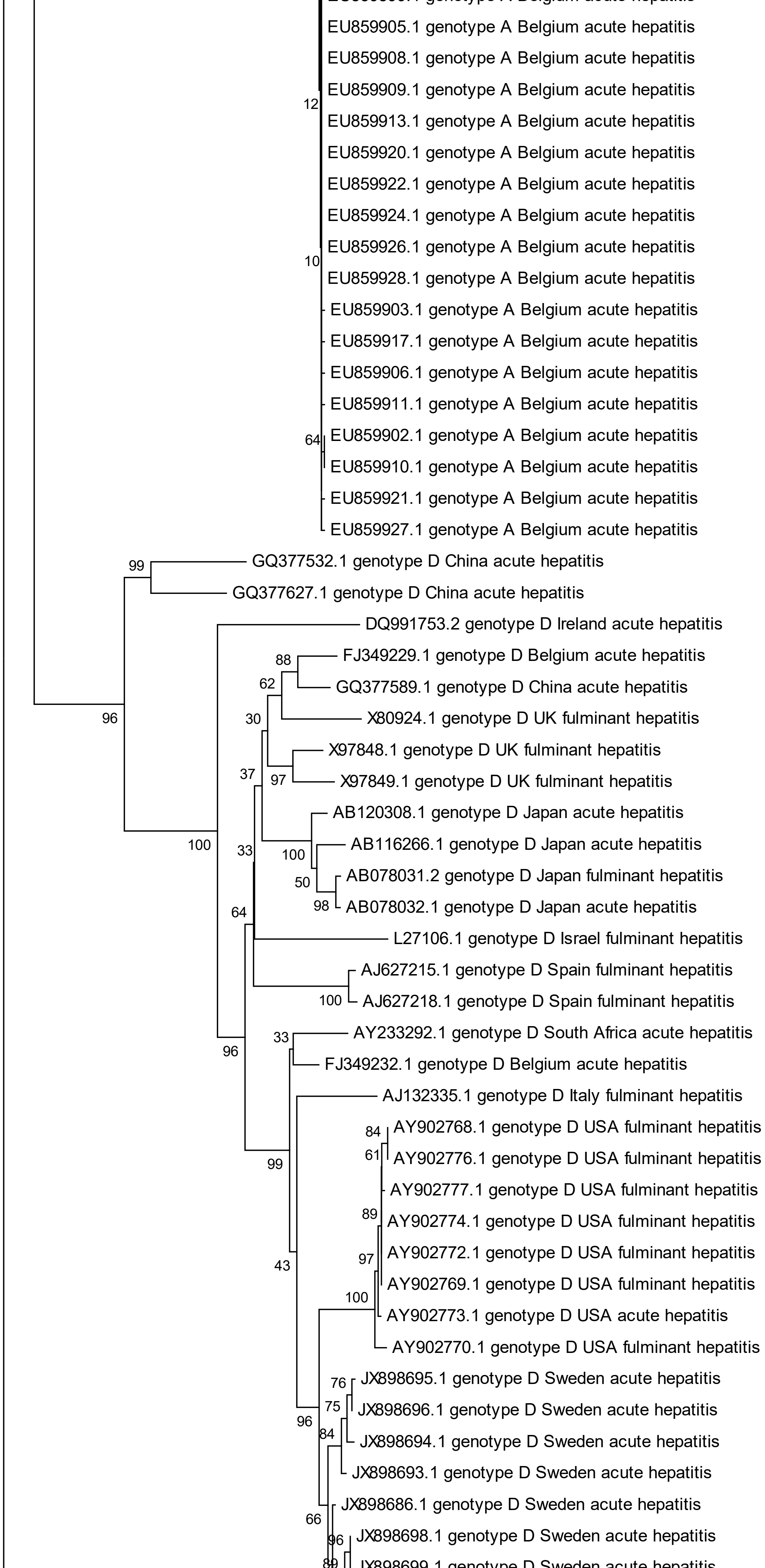

EU859905.1 genotype A Belgium acute hepatitis
EU859908.1 genotype A Belgium acute hepatitis
EU859909.1 genotype A Belgium acute hepatitis
12
EU859913.1 genotype A Belgium acute hepatitis
EU859920.1 genotype A Belgium acute hepatitis
EU859922.1 genotype A Belgium acute hepatitis
EU859924.1 genotype A Belgium acute hepatitis
EU859926.1 genotype A Belgium acute hepatitis
10
EU859928.1 genotype A Belgium acute hepatitis
EU859903.1 genotype A Belgium acute hepatitis
EU859917.1 genotype A Belgium acute hepatitis
EU859906.1 genotype A Belgium acute hepatitis
EU859911.1 genotype A Belgium acute hepatitis
64
EU859902.1 genotype A Belgium acute hepatitis
EU859910.1 genotype A Belgium acute hepatitis
EU859921.1 genotype A Belgium acute hepatitis
EU859927.1 genotype A Belgium acute hepatitis
99
GQ377532.1 genotype D China acute hepatitis
GQ377627.1 genotype D China acute hepatitis
DQ991753.2 genotype D Ireland acute hepatitis
88
FJ349229.1 genotype D Belgium acute hepatitis
62
GQ377589.1 genotype D China acute hepatitis
30
X80924.1 genotype D UK fulminant hepatitis
96
X97848.1 genotype D UK fulminant hepatitis
37
97
X97849.1 genotype D UK fulminant hepatitis
AB120308.1 genotype D Japan acute hepatitis
100
33
AB116266.1 genotype D Japan acute hepatitis
100
AB078031.2 genotype D Japan fulminant hepatitis
50
98
AB078032.1 genotype D Japan acute hepatitis
64
L27106.1 genotype D Israel fulminant hepatitis
AJ627215.1 genotype D Spain fulminant hepatitis
100
AJ627218.1 genotype D Spain fulminant hepatitis
33
AY233292.1 genotype D South Africa acute hepatitis
96
FJ349232.1 genotype D Belgium acute hepatitis
AJ132335.1 genotype D Italy fulminant hepatitis
84
AY902768.1 genotype D USA fulminant hepatitis
61
AY902776.1 genotype D USA fulminant hepatitis
99
AY902777.1 genotype D USA fulminant hepatitis
89
AY902774.1 genotype D USA fulminant hepatitis
97
AY902772.1 genotype D USA fulminant hepatitis
43
AY902769.1 genotype D USA fulminant hepatitis
100
AY902773.1 genotype D USA acute hepatitis
AY902770.1 genotype D USA fulminant hepatitis
76
JX898695.1 genotype D Sweden acute hepatitis
75
JX898696.1 genotype D Sweden acute hepatitis
96
84
JX898694.1 genotype D Sweden acute hepatitis
JX898693.1 genotype D Sweden acute hepatitis
JX898686.1 genotype D Sweden acute hepatitis
66
96
JX898698.1 genotype D Sweden acute hepatitis

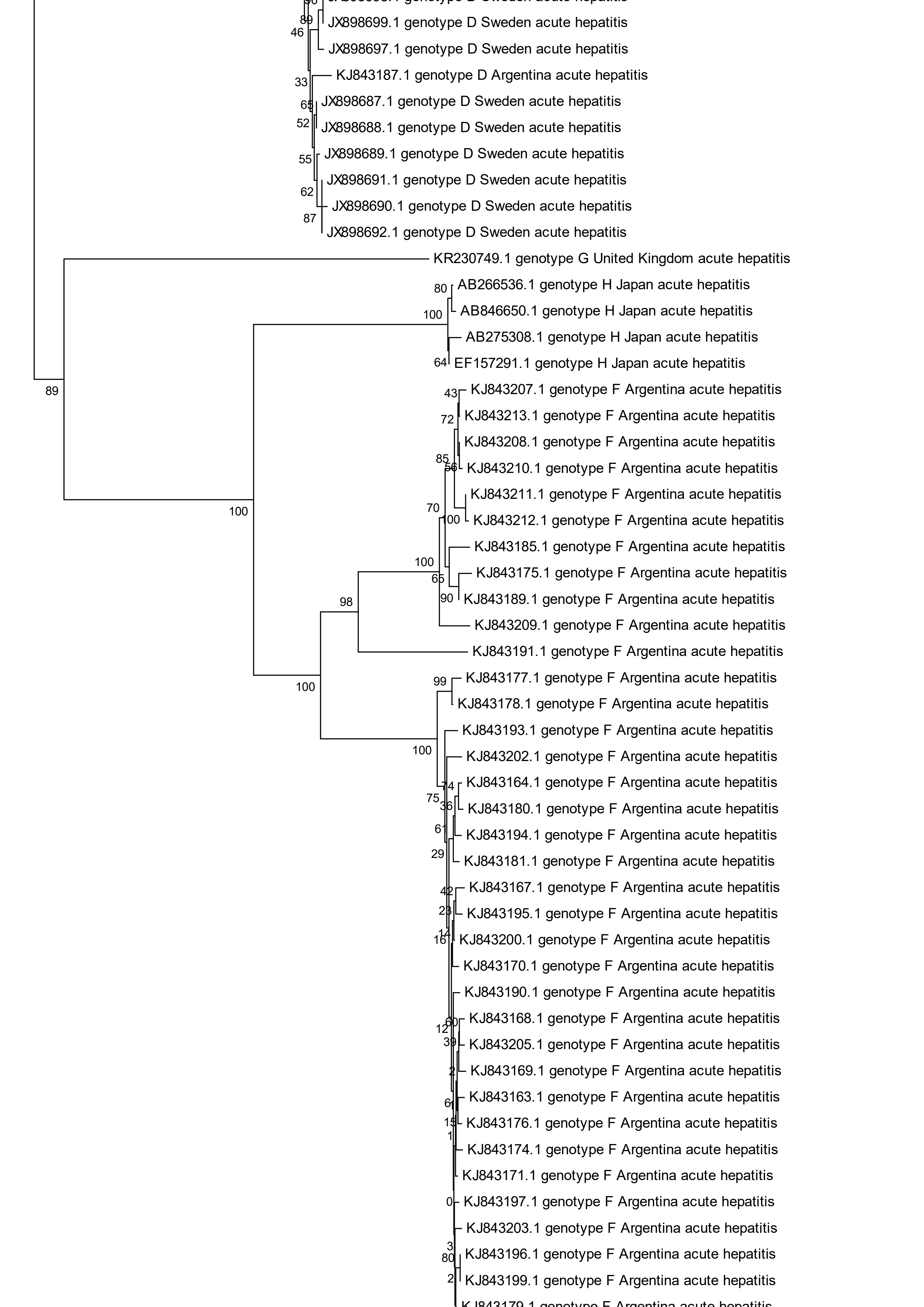

JX898699.1 genotype D Sweden acute hepatitis
JX898697.1 genotype D Sweden acute hepatitis
KJ843187.1 genotype D Argentina acute hepatitis
JX898687.1 genotype D Sweden acute hepatitis
JX898688.1 genotype D Sweden acute hepatitis
JX898689.1 genotype D Sweden acute hepatitis
JX898691.1 genotype D Sweden acute hepatitis
JX898690.1 genotype D Sweden acute hepatitis
JX898692.1 genotype D Sweden acute hepatitis
KR230749.1 genotype G United Kingdom acute hepatitis
AB266536.1 genotype H Japan acute hepatitis
AB846650.1 genotype H Japan acute hepatitis
AB275308.1 genotype H Japan acute hepatitis
EF157291.1 genotype H Japan acute hepatitis
KJ843207.1 genotype F Argentina acute hepatitis
KJ843213.1 genotype F Argentina acute hepatitis
KJ843208.1 genotype F Argentina acute hepatitis
KJ843210.1 genotype F Argentina acute hepatitis
KJ843211.1 genotype F Argentina acute hepatitis
KJ843212.1 genotype F Argentina acute hepatitis
KJ843185.1 genotype F Argentina acute hepatitis
KJ843175.1 genotype F Argentina acute hepatitis
KJ843189.1 genotype F Argentina acute hepatitis
KJ843209.1 genotype F Argentina acute hepatitis
KJ843191.1 genotype F Argentina acute hepatitis
KJ843177.1 genotype F Argentina acute hepatitis
KJ843178.1 genotype F Argentina acute hepatitis
KJ843193.1 genotype F Argentina acute hepatitis
KJ843202.1 genotype F Argentina acute hepatitis
KJ843164.1 genotype F Argentina acute hepatitis
KJ843180.1 genotype F Argentina acute hepatitis
KJ843194.1 genotype F Argentina acute hepatitis
KJ843181.1 genotype F Argentina acute hepatitis
KJ843167.1 genotype F Argentina acute hepatitis
KJ843195.1 genotype F Argentina acute hepatitis
KJ843200.1 genotype F Argentina acute hepatitis
KJ843170.1 genotype F Argentina acute hepatitis
KJ843190.1 genotype F Argentina acute hepatitis
KJ843168.1 genotype F Argentina acute hepatitis
KJ843205.1 genotype F Argentina acute hepatitis
KJ843169.1 genotype F Argentina acute hepatitis
KJ843163.1 genotype F Argentina acute hepatitis
KJ843176.1 genotype F Argentina acute hepatitis
KJ843174.1 genotype F Argentina acute hepatitis
KJ843171.1 genotype F Argentina acute hepatitis
KJ843197.1 genotype F Argentina acute hepatitis
KJ843203.1 genotype F Argentina acute hepatitis
KJ843196.1 genotype F Argentina acute hepatitis
KJ843199.1 genotype F Argentina acute hepatitis

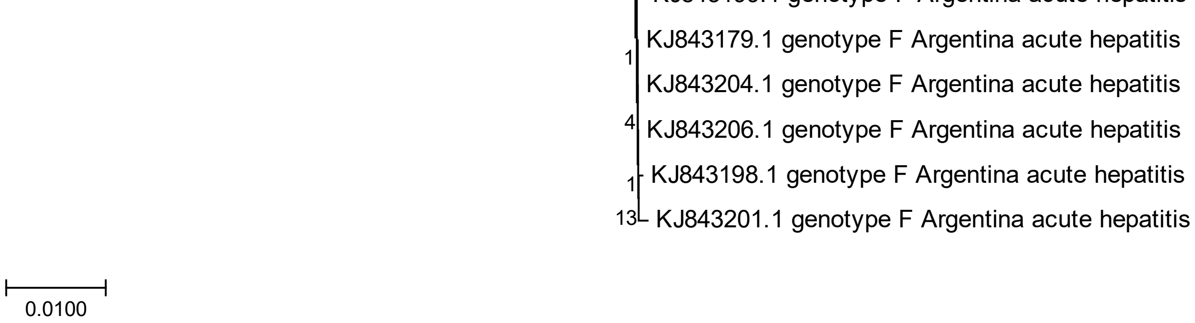
1
KJ843179.1 genotype F Argentina acute hepatitis
KJ843204.1 genotype F Argentina acute hepatitis
4
KJ843206.1 genotype F Argentina acute hepatitis
1
KJ843198.1 genotype F Argentina acute hepatitis
13
KJ843201.1 genotype F Argentina acute hepatitis
0.0100
