# Supplementary material for: Novel Genetic Variants of Hepatitis B Virus in Fulminant Hepatitis
Source: J Pathog. 2017 Dec 19;2017:1231204. doi: 10.1155/2017/1231204 (PMC5749291; doi:10.1155/2017/1231204)
Supplement: Supplementary 2 — Suppl Table 1: genotype and geographical distribution of fulminant and acute hepatitis B. [file 1231204.f2.pdf]

Accession Number	Genotype	Country	Hepatitis
AF090838.1	genotype A	France	subfulminant hepatitis
AF297621.1	genotype A	South Africa	fulminant hepatitis
AF297622.1	genotype A	South Africa	fulminant hepatitis
AF297623.1	genotype A	South Africa	fulminant hepatitis
AF297624.1	genotype A	South Africa	fulminant hepatitis
AF297625.1	genotype A	South Africa	fulminant hepatitis
AY233284.1	genotype A	South Africa	fulminant hepatitis
LC051141.1	genotype A	Japan	fulminant hepatitis
AB010290.1	genotype B	Japan	fulminant hepatitis
AB031266.1	genotype B	Vietnam	fulminant hepatitis
AB031267.1	genotype B	Vietnam	fulminant hepatitis
AB100695.1	genotype B	Vietnam	fulminant hepatitis
AB300370.1	genotype B	Japan	fulminant hepatitis
AB300371.1	genotype B	Japan	fulminant hepatitis
AB302942.1	genotype B	Japan	fulminant hepatitis
AB302943.1	genotype B	Japan	fulminant hepatitis
AB302944.1	genotype B	Japan	fulminant hepatitis
AB302945.1	genotype B	Japan	fulminant hepatitis
AB642091.1	genotype B	Japan	fulminant hepatitis
AB642092.1	genotype B	Japan	fulminant hepatitis
AB642093.1	genotype B	Japan	fulminant hepatitis
AB642094.1	genotype B	Japan	fulminant hepatitis
AB642098.1	genotype B	Japan	fulminant hepatitis
AB931168.1	genotype B	Japan	fulminant hepatitis
AF461360.1	genotype B	China	fulminant hepatitis
AF461362.1	genotype B	China	fulminant hepatitis
D50521.1	genotype B	Japan	fulminant hepatitis
D50522.1	genotype B	Japan	fulminant hepatitis
X97850.1	genotype B	UK	fulminant hepatitis
X97851.1	genotype B	UK	fulminant hepatitis
AB031265.1	genotype C	Vietnam	fulminant hepatitis
AB049609.1	genotype C	Japan	fulminant hepatitis
AB049610.1	genotype C	Japan	fulminant hepatitis
AB205152.1	genotype C	Japan	fulminant hepatitis
AB300368.1	genotype C	Japan	fulminant hepatitis
AB300369.1	genotype C	Japan	fulminant hepatitis
AB300372.1	genotype C	Japan	fulminant hepatitis
AB300373.1	genotype C	Japan	fulminant hepatitis
AB642095.1	genotype C	Japan	fulminant hepatitis
AB642096.1	genotype C	Japan	fulminant hepatitis
AB642097.1	genotype C	Japan	fulminant hepatitis
AB642099.1	genotype C	Japan	fulminant hepatitis
AB642100.1	genotype C	Japan	fulminant hepatitis
AB931170.1	genotype C	Japan	fulminant hepatitis
AF458664.1	genotype C	China	fulminant hepatitis
AF458665.1	genotype C	China	fulminant hepatitis
AF461357.1	genotype C	China	fulminant hepatitis
AF461358.1	genotype C	China	fulminant hepatitis
AF461361.1	genotype C	China	fulminant hepatitis

Accession Number	Genotype	Country	Hepatitis
AY066028.1	genotype C	China	fulminant hepatitis
AY306136.1	genotype C	China	fulminant hepatitis
GQ872211.1	genotype C	South Korea	fulminant hepatitis
AB078031.2	genotype D	Japan	fulminant hepatitis
AJ132335.1	genotype D	Italy	fulminant hepatitis
AJ627215.1	genotype D	Spain	fulminant hepatitis
AJ627218.1	genotype D	Spain	fulminant hepatitis
AY902768.1	genotype D	USA	fulminant hepatitis
AY902769.1	genotype D	USA	fulminant hepatitis
AY902770.1	genotype D	USA	fulminant hepatitis
AY902772.1	genotype D	USA	fulminant hepatitis
AY902774.1	genotype D	USA	fulminant hepatitis
AY902776.1	genotype D	USA	fulminant hepatitis
AY902777.1	genotype D	USA	fulminant hepatitis
L27106.1	genotype D	Israel	fulminant hepatitis
X80924.1	genotype D	UK	fulminant hepatitis
X97848.1	genotype D	UK	fulminant hepatitis
X97849.1	genotype D	UK	fulminant hepatitis
AB300366.1	genotype A	Japan	acute hepatitis
AB300367.1	genotype A	Japan	acute hepatitis
AB775198.1	genotype A	Japan	acute hepatitis
AB775199.1	genotype A	Japan	acute hepatitis
AB775200.1	genotype A	Japan	acute hepatitis
AB775201.1	genotype A	Japan	acute hepatitis
AB778116.1	genotype A	Japan	acute hepatitis
AB937791.1	genotype A	Japan	acute hepatitis
AB937792.1	genotype A	Japan	acute hepatitis
AB937793.1	genotype A	Japan	acute hepatitis
AB937794.1	genotype A	Japan	acute hepatitis
AY233274.1	genotype A	South Africa	acute hepatitis
AY233279.1	genotype A	South Africa	acute hepatitis
AY233283.1	genotype A	South Africa	acute hepatitis
AY233287.1	genotype A	South Africa	acute hepatitis
EU859898.1	genotype A	Belgium	acute hepatitis
EU859899.1	genotype A	Belgium	acute hepatitis
EU859900.1	genotype A	Belgium	acute hepatitis
EU859901.1	genotype A	Belgium	acute hepatitis
EU859902.1	genotype A	Belgium	acute hepatitis
EU859903.1	genotype A	Belgium	acute hepatitis
EU859904.1	genotype A	Belgium	acute hepatitis
EU859905.1	genotype A	Belgium	acute hepatitis
EU859906.1	genotype A	Belgium	acute hepatitis
EU859907.1	genotype A	Belgium	acute hepatitis
EU859908.1	genotype A	Belgium	acute hepatitis
EU859909.1	genotype A	Belgium	acute hepatitis
EU859910.1	genotype A	Belgium	acute hepatitis
EU859911.1	genotype A	Belgium	acute hepatitis
EU859912.1	genotype A	Belgium	acute hepatitis
EU859913.1	genotype A	Belgium	acute hepatitis

Accession Number	Genotype	Country	Hepatitis
EU859914.1	genotype A	Belgium	acute hepatitis
EU859915.1	genotype A	Belgium	acute hepatitis
EU859916.1	genotype A	Belgium	acute hepatitis
EU859917.1	genotype A	Belgium	acute hepatitis
EU859918.1	genotype A	Belgium	acute hepatitis
EU859919.1	genotype A	Belgium	acute hepatitis
EU859920.1	genotype A	Belgium	acute hepatitis
EU859921.1	genotype A	Belgium	acute hepatitis
EU859922.1	genotype A	Belgium	acute hepatitis
EU859923.1	genotype A	Belgium	acute hepatitis
EU859924.1	genotype A	Belgium	acute hepatitis
EU859925.1	genotype A	Belgium	acute hepatitis
EU859926.1	genotype A	Belgium	acute hepatitis
EU859927.1	genotype A	Belgium	acute hepatitis
EU859928.1	genotype A	Belgium	acute hepatitis
KC836877.1	genotype A	Japan	acute hepatitis
KC836878.1	genotype A	Japan	acute hepatitis
KC836879.1	genotype A	Japan	acute hepatitis
KC836880.1	genotype A	Japan	acute hepatitis
KC836881.1	genotype A	Japan	acute hepatitis
KJ843166.1	genotype A	Argentina	acute hepatitis
KJ843172.1	genotype A	Argentina	acute hepatitis
KJ843173.1	genotype A	Argentina	acute hepatitis
KJ843182.1	genotype A	Argentina	acute hepatitis
KJ843183.1	genotype A	Argentina	acute hepatitis
KJ843184.1	genotype A	Argentina	acute hepatitis
KJ843186.1	genotype A	Argentina	acute hepatitis
KJ843188.1	genotype A	Argentina	acute hepatitis
KJ843192.1	genotype A	Argentina	acute hepatitis
KJ843214.1	genotype A	Argentina	acute hepatitis
KJ843215.1	genotype A	Argentina	acute hepatitis
KJ843216.1	genotype A	Argentina	acute hepatitis
KJ843217.1	genotype A	Argentina	acute hepatitis
KJ843218.1	genotype A	Argentina	acute hepatitis
AB300364.1	genotype B	Japan	acute hepatitis
AB602818.1	genotype B	Japan	acute hepatitis
GQ377519.1	genotype B	China	acute hepatitis
GQ377525.1	genotype B	China	acute hepatitis
GQ377537.1	genotype B	China	acute hepatitis
GQ377542.1	genotype B	China	acute hepatitis
GQ377547.1	genotype B	China	acute hepatitis
GQ377550.1	genotype B	China	acute hepatitis
GQ377558.1	genotype B	China	acute hepatitis
GQ377561.1	genotype B	China	acute hepatitis
GQ377566.1	genotype B	China	acute hepatitis
GQ377567.1	genotype B	China	acute hepatitis
GQ377568.1	genotype B	China	acute hepatitis
GQ377569.1	genotype B	China	acute hepatitis
GQ377582.1	genotype B	China	acute hepatitis

Accession Number	Genotype	Country	Hepatitis
GQ377587.1	genotype B	China	acute hepatitis
GQ377588.1	genotype B	China	acute hepatitis
GQ377595.1	genotype B	China	acute hepatitis
GQ377606.1	genotype B	China	acute hepatitis
GQ377610.1	genotype B	China	acute hepatitis
GQ377612.1	genotype B	China	acute hepatitis
GQ377622.1	genotype B	China	acute hepatitis
GQ377625.1	genotype B	China	acute hepatitis
GQ377629.1	genotype B	China	acute hepatitis
GQ377638.1	genotype B	China	acute hepatitis
GQ377639.1	genotype B	China	acute hepatitis
GQ377641.1	genotype B	China	acute hepatitis
GQ377643.1	genotype B	China	acute hepatitis
GQ377644.1	genotype B	China	acute hepatitis
KJ843165.1	genotype B	Argentina	acute hepatitis
LC036263.1	genotype B	Japan	acute hepatitis
AB031262.1	genotype C	Vietnam	acute hepatitis
AB113879.1	genotype C	Japan	acute hepatitis
AB298720.1	genotype C	Japan	acute hepatitis
AB299858.1	genotype C	Japan	acute hepatitis
AB300359.1	genotype C	Japan	acute hepatitis
AB300360.1	genotype C	Japan	acute hepatitis
AB300361.1	genotype C	Japan	acute hepatitis
AB300362.1	genotype C	Japan	acute hepatitis
AB300363.1	genotype C	Japan	acute hepatitis
AB300365.1	genotype C	Japan	acute hepatitis
AB971715.1	genotype C	Japan	acute hepatitis
GQ377514.1	genotype C	China	acute hepatitis
GQ377515.1	genotype C	China	acute hepatitis
GQ377516.1	genotype C	China	acute hepatitis
GQ377517.1	genotype C	China	acute hepatitis
GQ377518.1	genotype C	China	acute hepatitis
GQ377520.1	genotype C	China	acute hepatitis
GQ377521.1	genotype C	China	acute hepatitis
GQ377522.1	genotype C	China	acute hepatitis
GQ377523.1	genotype C	China	acute hepatitis
GQ377524.1	genotype C	China	acute hepatitis
GQ377526.1	genotype C	China	acute hepatitis
GQ377527.1	genotype C	China	acute hepatitis
GQ377528.1	genotype C	China	acute hepatitis
GQ377529.1	genotype C	China	acute hepatitis
GQ377530.1	genotype C	China	acute hepatitis
GQ377531.1	genotype C	China	acute hepatitis
GQ377533.1	genotype C	China	acute hepatitis
GQ377534.1	genotype C	China	acute hepatitis
GQ377535.1	genotype C	China	acute hepatitis
GQ377536.1	genotype C	China	acute hepatitis
GQ377538.1	genotype C	China	acute hepatitis
GQ377539.1	genotype C	China	acute hepatitis

Accession Number	Genotype	Country	Hepatitis
GQ377540.1	genotype C	China	acute hepatitis
GQ377541.1	genotype C	China	acute hepatitis
GQ377543.1	genotype C	China	acute hepatitis
GQ377544.1	genotype C	China	acute hepatitis
GQ377545.1	genotype C	China	acute hepatitis
GQ377546.1	genotype C	China	acute hepatitis
GQ377548.1	genotype C	China	acute hepatitis
GQ377549.1	genotype C	China	acute hepatitis
GQ377551.1	genotype C	China	acute hepatitis
GQ377552.1	genotype C	China	acute hepatitis
GQ377553.1	genotype C	China	acute hepatitis
GQ377554.1	genotype C	China	acute hepatitis
GQ377555.1	genotype C	China	acute hepatitis
GQ377556.1	genotype C	China	acute hepatitis
GQ377557.1	genotype C	China	acute hepatitis
GQ377559.1	genotype C	China	acute hepatitis
GQ377560.1	genotype C	China	acute hepatitis
GQ377562.1	genotype C	China	acute hepatitis
GQ377563.1	genotype C	China	acute hepatitis
GQ377564.1	genotype C	China	acute hepatitis
GQ377565.1	genotype C	China	acute hepatitis
GQ377570.1	genotype C	China	acute hepatitis
GQ377571.1	genotype C	China	acute hepatitis
GQ377572.1	genotype C	China	acute hepatitis
GQ377573.1	genotype C	China	acute hepatitis
GQ377574.1	genotype C	China	acute hepatitis
GQ377575.1	genotype C	China	acute hepatitis
GQ377576.1	genotype C	China	acute hepatitis
GQ377577.1	genotype C	China	acute hepatitis
GQ377578.1	genotype C	China	acute hepatitis
GQ377579.1	genotype C	China	acute hepatitis
GQ377580.1	genotype C	China	acute hepatitis
GQ377581.1	genotype C	China	acute hepatitis
GQ377583.1	genotype C	China	acute hepatitis
GQ377584.1	genotype C	China	acute hepatitis
GQ377585.1	genotype C	China	acute hepatitis
GQ377586.1	genotype C	China	acute hepatitis
GQ377590.1	genotype C	China	acute hepatitis
GQ377591.1	genotype C	China	acute hepatitis
GQ377592.1	genotype C	China	acute hepatitis
GQ377593.1	genotype C	China	acute hepatitis
GQ377594.1	genotype C	China	acute hepatitis
GQ377596.1	genotype C	China	acute hepatitis
GQ377597.1	genotype C	China	acute hepatitis
GQ377598.1	genotype C	China	acute hepatitis
GQ377599.1	genotype C	China	acute hepatitis
GQ377600.1	genotype C	China	acute hepatitis
GQ377601.1	genotype C	China	acute hepatitis
GQ377602.1	genotype C	China	acute hepatitis

Accession Number	Genotype	Country	Hepatitis
GQ377603.1	genotype C	China	acute hepatitis
GQ377604.1	genotype C	China	acute hepatitis
GQ377605.1	genotype C	China	acute hepatitis
GQ377607.1	genotype C	China	acute hepatitis
GQ377608.1	genotype C	China	acute hepatitis
GQ377609.1	genotype C	China	acute hepatitis
GQ377611.1	genotype C	China	acute hepatitis
GQ377613.1	genotype C	China	acute hepatitis
GQ377614.1	genotype C	China	acute hepatitis
GQ377615.1	genotype C	China	acute hepatitis
GQ377616.1	genotype C	China	acute hepatitis
GQ377617.1	genotype C	China	acute hepatitis
GQ377618.1	genotype C	China	acute hepatitis
GQ377619.1	genotype C	China	acute hepatitis
GQ377620.1	genotype C	China	acute hepatitis
GQ377621.1	genotype C	China	acute hepatitis
GQ377623.1	genotype C	China	acute hepatitis
GQ377624.1	genotype C	China	acute hepatitis
GQ377626.1	genotype C	China	acute hepatitis
GQ377628.1	genotype C	China	acute hepatitis
GQ377630.1	genotype C	China	acute hepatitis
GQ377631.1	genotype C	China	acute hepatitis
GQ377632.1	genotype C	China	acute hepatitis
GQ377633.1	genotype C	China	acute hepatitis
GQ377634.1	genotype C	China	acute hepatitis
GQ377635.1	genotype C	China	acute hepatitis
GQ377636.1	genotype C	China	acute hepatitis
GQ377637.1	genotype C	China	acute hepatitis
GQ377640.1	genotype C	China	acute hepatitis
GQ377642.1	genotype C	China	acute hepatitis
AB078032.1	genotype D	Japan	acute hepatitis
AB116266.1	genotype D	Japan	acute hepatitis
AB120308.1	genotype D	Japan	acute hepatitis
AY233292.1	genotype D	South Africa	acute hepatitis
AY902773.1	genotype D	USA	acute hepatitis
DQ991753.2	genotype D	Ireland	acute hepatitis
FJ349229.1	genotype D	Belgium	acute hepatitis
FJ349232.1	genotype D	Belgium	acute hepatitis
GQ377532.1	genotype D	China	acute hepatitis
GQ377589.1	genotype D	China	acute hepatitis
GQ377627.1	genotype D	China	acute hepatitis
JX898686.1	genotype D	Sweden	acute hepatitis
JX898687.1	genotype D	Sweden	acute hepatitis
JX898688.1	genotype D	Sweden	acute hepatitis
JX898689.1	genotype D	Sweden	acute hepatitis
JX898690.1	genotype D	Sweden	acute hepatitis
JX898691.1	genotype D	Sweden	acute hepatitis
JX898692.1	genotype D	Sweden	acute hepatitis
JX898693.1	genotype D	Sweden	acute hepatitis

Accession Number	Genotype	Country	Hepatitis
JX898694.1	genotype D	Sweden	acute hepatitis
JX898695.1	genotype D	Sweden	acute hepatitis
JX898696.1	genotype D	Sweden	acute hepatitis
JX898697.1	genotype D	Sweden	acute hepatitis
JX898698.1	genotype D	Sweden	acute hepatitis
JX898699.1	genotype D	Sweden	acute hepatitis
KJ843187.1	genotype D	Argentina	acute hepatitis
KJ843163.1	genotype F	Argentina	acute hepatitis
KJ843164.1	genotype F	Argentina	acute hepatitis
KJ843167.1	genotype F	Argentina	acute hepatitis
KJ843168.1	genotype F	Argentina	acute hepatitis
KJ843169.1	genotype F	Argentina	acute hepatitis
KJ843170.1	genotype F	Argentina	acute hepatitis
KJ843171.1	genotype F	Argentina	acute hepatitis
KJ843174.1	genotype F	Argentina	acute hepatitis
KJ843175.1	genotype F	Argentina	acute hepatitis
KJ843176.1	genotype F	Argentina	acute hepatitis
KJ843177.1	genotype F	Argentina	acute hepatitis
KJ843178.1	genotype F	Argentina	acute hepatitis
KJ843179.1	genotype F	Argentina	acute hepatitis
KJ843180.1	genotype F	Argentina	acute hepatitis
KJ843181.1	genotype F	Argentina	acute hepatitis
KJ843185.1	genotype F	Argentina	acute hepatitis
KJ843189.1	genotype F	Argentina	acute hepatitis
KJ843190.1	genotype F	Argentina	acute hepatitis
KJ843191.1	genotype F	Argentina	acute hepatitis
KJ843193.1	genotype F	Argentina	acute hepatitis
KJ843194.1	genotype F	Argentina	acute hepatitis
KJ843195.1	genotype F	Argentina	acute hepatitis
KJ843196.1	genotype F	Argentina	acute hepatitis
KJ843197.1	genotype F	Argentina	acute hepatitis
KJ843198.1	genotype F	Argentina	acute hepatitis
KJ843199.1	genotype F	Argentina	acute hepatitis
KJ843200.1	genotype F	Argentina	acute hepatitis
KJ843201.1	genotype F	Argentina	acute hepatitis
KJ843202.1	genotype F	Argentina	acute hepatitis
KJ843203.1	genotype F	Argentina	acute hepatitis
KJ843204.1	genotype F	Argentina	acute hepatitis
KJ843205.1	genotype F	Argentina	acute hepatitis
KJ843206.1	genotype F	Argentina	acute hepatitis
KJ843207.1	genotype F	Argentina	acute hepatitis
KJ843208.1	genotype F	Argentina	acute hepatitis
KJ843209.1	genotype F	Argentina	acute hepatitis
KJ843210.1	genotype F	Argentina	acute hepatitis
KJ843211.1	genotype F	Argentina	acute hepatitis
KJ843212.1	genotype F	Argentina	acute hepatitis
KJ843213.1	genotype F	Argentina	acute hepatitis
KR230749.1	genotype G	UK	acute hepatitis
AB266536.1	genotype H	Japan	acute hepatitis

Accession Number	Genotype	Country	Hepatitis
AB275308.1	genotype H	Japan	acute hepatitis
AB846650.1	genotype H	Japan	acute hepatitis
EF157291.1	genotype H	Japan	acute hepatitis
AB231908.1	genotype I	Vietnam	acute hepatitis
