# Supplementary material for: Novel Genetic Variants of Hepatitis B Virus in Fulminant Hepatitis
Source: J Pathog. 2017 Dec 19;2017:1231204. doi: 10.1155/2017/1231204 (PMC5749291; doi:10.1155/2017/1231204)
Supplement: Supplementary 3 — Suppl Table 2: top 55 candidate nucleotides associated with fulminant hepatitis B. [file 1231204.f3.pdf]

**Suppl Table 2** Top 55 candidate nucleotides associated with fulminant hepatitis B

| Nucleotide | Fulminant, n = 67 (%sensitivity) | Acute,[a] n = 280 (%specificity) | %Accuracy[b] |
|---|---|---|---|
| A1896 | 41 (61.2) | 24 (91.4) | 297 (85.6) |
| C2129 | 17 (25.4) | 1 (99.6) | 296 (85.3) |
| A2345 | 17 (25.4) | 3 (98.9) | 294 (84.7) |
| G2003 | 16 (23.9) | 4 (98.6) | 292 (84.1) |
| C2441 | 20 (29.9) | 9 (96.8) | 291 (83.9) |
| G1981 | 10 (14.9) | 0 (100.0) | 290 (83.6) |
| T2755 | 11 (16.4) | 1 (99.6) | 290 (83.6) |
| A2136 | 13 (19.4) | 3 (98.9) | 290 (83.6) |
| B3106 | 10 (14.9) | 1 (99.6) | 289 (83.3) |
| C53 | 15 (22.4) | 6 (97.9) | 289 (83.3) |
| C1961 | 8 (11.9) | 0 (100.0) | 288 (83.0) |
| T720 | 9 (13.4) | 1 (99.6) | 288 (83.0) |
| G2430 | 9 (13.4) | 1 (99.6) | 288 (83.0) |
| G2431 | 9 (13.4) | 1 (99.6) | 288 (83.0) |
| D2303 | 10 (14.9) | 2 (99.3) | 288 (83.0) |
| C3013 | 11 (16.4) | 3 (98.9) | 288 (83.0) |
| S481 | 8 (11.9) | 1 (99.6) | 287 (82.7) |
| G1838 | 8 (11.9) | 1 (99.6) | 287 (82.7) |
| D1962 | 8 (11.9) | 1 (99.6) | 287 (82.7) |
| G273 | 9 (13.4) | 2 (99.3) | 287 (82.7) |
| M2546 | 10 (14.9) | 3 (98.9) | 287 (82.7) |
| C289 | 12 (17.9) | 5 (98.2) | 287 (82.7) |
| K2339 | 12 (17.9) | 5 (98.2) | 287 (82.7) |
| A2597 | 12 (17.9) | 5 (98.2) | 287 (82.7) |
| T1653 | 20 (29.9) | 13 (95.4) | 287 (82.7) |
| A1410 | 6 (9.0) | 0 (100.0) | 286 (82.4) |
| Y2092 | 6 (9.0) | 0 (100.0) | 286 (82.4) |

| | | | |
|---|---|---|---|
| Y2131 | 6 (9.0) | 0 (100.0) | 286 (82.4) |
| A2222 | 6 (9.0) | 0 (100.0) | 286 (82.4) |
| C280 | 7 (10.4) | 1 (99.6) | 286 (82.4) |
| C774 | 7 (10.4) | 1 (99.6) | 286 (82.4) |
| G2140 | 7 (10.4) | 1 (99.6) | 286 (82.4) |
| G2173 | 7 (10.4) | 1 (99.6) | 286 (82.4) |
| T2979 | 7 (10.4) | 1 (99.6) | 286 (82.4) |
| G300 | 8 (11.9) | 2 (99.3) | 286 (82.4) |
| W1739 | 8 (11.9) | 2 (99.3) | 286 (82.4) |
| G2340 | 9 (13.4) | 3 (98.9) | 286 (82.4) |
| R2522 | 9 (13.4) | 3 (98.9) | 286 (82.4) |
| S2708 | 9 (13.4) | 3 (98.9) | 286 (82.4) |
| C123 | 5 (7.5) | 0 (100.0) | 285 (82.1) |
| C777 | 5 (7.5) | 0 (100.0) | 285 (82.1) |
| A787 | 5 (7.5) | 0 (100.0) | 285 (82.1) |
| T2013 | 5 (7.5) | 0 (100.0) | 285 (82.1) |
| K2048 | 5 (7.5) | 0 (100.0) | 285 (82.1) |
| A2512 | 5 (7.5) | 0 (100.0) | 285 (82.1) |
| A3088 | 5 (7.5) | 0 (100.0) | 285 (82.1) |
| C132 | 6 (9.0) | 1 (99.6) | 285 (82.1) |
| T343 | 6 (9.0) | 1 (99.6) | 285 (82.1) |
| W1677 | 6 (9.0) | 1 (99.6) | 285 (82.1) |
| A1739 | 7 (10.4) | 2 (99.3) | 285 (82.1) |
| C2489 | 7 (10.4) | 2 (99.3) | 285 (82.1) |
| G2150 | 8 (11.9) | 3 (98.9) | 285 (82.1) |
| C2151 | 8 (11.9) | 3 (98.9) | 285 (82.1) |
| A2410 | 8 (11.9) | 3 (98.9) | 285 (82.1) |
| A2636 | 10 (14.9) | 5 (98.2) | 285 (82.1) |

SVM, support vector machine.
[a]Acute indicates acute non-fulminant hepatitis cases.
[b]% Accuracy = (67 x %sensitivity + 280 x %specificity)/(67 + 280).
