# Supplementary material for: Novel Genetic Variants of Hepatitis B Virus in Fulminant Hepatitis
Source: J Pathog. 2017 Dec 19;2017:1231204. doi: 10.1155/2017/1231204 (PMC5749291; doi:10.1155/2017/1231204)
Supplement: Supplementary 4 — Suppl Table 3: list of top 25 most informative nucleotide variants associated with fulminant hepatitis B in combinations generated using SVM modelling with brute force selection. [file 1231204.f4.pdf]

Index	Features	SNP1	SNP2	SNP3	SNP4	SNP5	SNP6	accuracy	sensitivity	specificity	PPV	NPV
143694	6.00	C2129	T720	Y2131	T2013	K2048	A2512	92.51%	64.18%	99.29%	95.56%	92.05%
144114	6.00	C2129	T720	Y2092	Y2131	K2048	A2512	92.51%	64.18%	99.29%	95.56%	92.05%
135493	6.00	C2129	Y2131	T2979	T2013	K2048	A2512	92.22%	62.69%	99.29%	95.45%	91.75%
135549	6.00	C2129	Y2131	G2173	T2013	K2048	A2512	92.22%	62.69%	99.29%	95.45%	91.75%
135633	6.00	C2129	Y2131	C774	T2013	K2048	A2512	92.22%	62.69%	99.29%	95.45%	91.75%
135753	6.00	C2129	Y2131	C280	T2013	K2048	A2512	92.22%	62.69%	99.29%	95.45%	91.75%
136449	6.00	C2129	Y2092	Y2131	T2979	K2048	A2512	92.22%	62.69%	99.29%	95.45%	91.75%
136477	6.00	C2129	Y2092	Y2131	G2173	K2048	A2512	92.22%	62.69%	99.29%	95.45%	91.75%
136513	6.00	C2129	Y2092	Y2131	C774	K2048	A2512	92.22%	62.69%	99.29%	95.45%	91.75%
136558	6.00	C2129	Y2092	Y2131	C280	K2048	A2512	92.22%	62.69%	99.29%	95.45%	91.75%
137164	6.00	C2129	A1410	Y2131	T2979	K2048	A2512	92.22%	62.69%	99.29%	95.45%	91.75%
137192	6.00	C2129	A1410	Y2131	G2173	K2048	A2512	92.22%	62.69%	99.29%	95.45%	91.75%
137228	6.00	C2129	A1410	Y2131	C774	K2048	A2512	92.22%	62.69%	99.29%	95.45%	91.75%
137273	6.00	C2129	A1410	Y2131	C280	K2048	A2512	92.22%	62.69%	99.29%	95.45%	91.75%
139494	6.00	C2129	G2431	Y2131	T2013	K2048	A2512	92.22%	62.69%	99.29%	95.45%	91.75%
139914	6.00	C2129	G2431	Y2092	Y2131	K2048	A2512	92.22%	62.69%	99.29%	95.45%	91.75%
140200	6.00	C2129	G2431	A1410	Y2131	K2048	A2512	92.22%	62.69%	99.29%	95.45%	91.75%
141314	6.00	C2129	G2430	Y2131	T2013	K2048	A2512	92.22%	62.69%	99.29%	95.45%	91.75%
141734	6.00	C2129	G2430	Y2092	Y2131	K2048	A2512	92.22%	62.69%	99.29%	95.45%	91.75%
142020	6.00	C2129	G2430	A1410	Y2131	K2048	A2512	92.22%	62.69%	99.29%	95.45%	91.75%
143677	6.00	C2129	T720	Y2131	K2048	A2512	W1677	92.22%	64.18%	98.93%	93.48%	92.03%
143687	6.00	C2129	T720	Y2131	T2013	A2512	W1677	92.22%	64.18%	98.93%	93.48%	92.03%
143715	6.00	C2129	T720	Y2131	C123	T2013	K2048	92.22%	62.69%	99.29%	95.45%	91.75%
144107	6.00	C2129	T720	Y2092	Y2131	A2512	W1677	92.22%	64.18%	98.93%	93.48%	92.03%
144126	6.00	C2129	T720	Y2092	Y2131	C123	K2048	92.22%	62.69%	99.29%	95.45%	91.75%
144400	6.00	C2129	T720	A1410	Y2131	K2048	A2512	92.22%	62.69%	99.29%	95.45%	91.75%
150630	6.00	C2129	T2755	Y2131	T2013	K2048	A2512	92.22%	62.69%	99.29%	95.45%	91.75%
151050	6.00	C2129	T2755	Y2092	Y2131	K2048	A2512	92.22%	62.69%	99.29%	95.45%	91.75%
151336	6.00	C2129	T2755	A1410	Y2131	K2048	A2512	92.22%	62.69%	99.29%	95.45%	91.75%
158040	6.00	C2129	G1981	T720	T2013	K2048	A2512	92.22%	62.69%	99.29%	95.45%	91.75%
158306	6.00	C2129	G1981	T720	Y2092	K2048	A2512	92.22%	62.69%	99.29%	95.45%	91.75%
135476	6.00	C2129	Y2131	T2979	K2048	A2512	W1677	91.93%	62.69%	98.93%	93.33%	91.72%

Index	Features	SNP1	SNP2	SNP3	SNP4	SNP5	SNP6	accuracy	sensitivity	specificity	PPV	NPV
135486	6.00	C2129	Y2131	T2979	T2013	A2512	W1677	91.93%	62.69%	98.93%	93.33%	91.72%
135514	6.00	C2129	Y2131	T2979	C123	T2013	K2048	91.93%	61.19%	99.29%	95.35%	91.45%
135532	6.00	C2129	Y2131	G2173	K2048	A2512	W1677	91.93%	62.69%	98.93%	93.33%	91.72%
135542	6.00	C2129	Y2131	G2173	T2013	A2512	W1677	91.93%	62.69%	98.93%	93.33%	91.72%
135570	6.00	C2129	Y2131	G2173	C123	T2013	K2048	91.93%	61.19%	99.29%	95.35%	91.45%
135616	6.00	C2129	Y2131	C774	K2048	A2512	W1677	91.93%	62.69%	98.93%	93.33%	91.72%
135626	6.00	C2129	Y2131	C774	T2013	A2512	W1677	91.93%	62.69%	98.93%	93.33%	91.72%
135654	6.00	C2129	Y2131	C774	C123	T2013	K2048	91.93%	61.19%	99.29%	95.35%	91.45%
135736	6.00	C2129	Y2131	C280	K2048	A2512	W1677	91.93%	62.69%	98.93%	93.33%	91.72%
135746	6.00	C2129	Y2131	C280	T2013	A2512	W1677	91.93%	62.69%	98.93%	93.33%	91.72%
135774	6.00	C2129	Y2131	C280	C123	T2013	K2048	91.93%	61.19%	99.29%	95.35%	91.45%
136442	6.00	C2129	Y2092	Y2131	T2979	A2512	W1677	91.93%	62.69%	98.93%	93.33%	91.72%
136461	6.00	C2129	Y2092	Y2131	T2979	C123	K2048	91.93%	61.19%	99.29%	95.35%	91.45%
136470	6.00	C2129	Y2092	Y2131	G2173	A2512	W1677	91.93%	62.69%	98.93%	93.33%	91.72%
136489	6.00	C2129	Y2092	Y2131	G2173	C123	K2048	91.93%	61.19%	99.29%	95.35%	91.45%
136506	6.00	C2129	Y2092	Y2131	C774	A2512	W1677	91.93%	62.69%	98.93%	93.33%	91.72%
136525	6.00	C2129	Y2092	Y2131	C774	C123	K2048	91.93%	61.19%	99.29%	95.35%	91.45%
136551	6.00	C2129	Y2092	Y2131	C280	A2512	W1677	91.93%	62.69%	98.93%	93.33%	91.72%
136570	6.00	C2129	Y2092	Y2131	C280	C123	K2048	91.93%	61.19%	99.29%	95.35%	91.45%
137157	6.00	C2129	A1410	Y2131	T2979	A2512	W1677	91.93%	62.69%	98.93%	93.33%	91.72%
137176	6.00	C2129	A1410	Y2131	T2979	C123	K2048	91.93%	61.19%	99.29%	95.35%	91.45%
137185	6.00	C2129	A1410	Y2131	G2173	A2512	W1677	91.93%	62.69%	98.93%	93.33%	91.72%
137204	6.00	C2129	A1410	Y2131	G2173	C123	K2048	91.93%	61.19%	99.29%	95.35%	91.45%
137221	6.00	C2129	A1410	Y2131	C774	A2512	W1677	91.93%	62.69%	98.93%	93.33%	91.72%
137240	6.00	C2129	A1410	Y2131	C774	C123	K2048	91.93%	61.19%	99.29%	95.35%	91.45%
137266	6.00	C2129	A1410	Y2131	C280	A2512	W1677	91.93%	62.69%	98.93%	93.33%	91.72%
137285	6.00	C2129	A1410	Y2131	C280	C123	K2048	91.93%	61.19%	99.29%	95.35%	91.45%
138122	6.00	C2129	D2303	Y2131	T2013	A2512	W1677	91.93%	64.18%	98.57%	91.49%	92.00%
138129	6.00	C2129	D2303	Y2131	T2013	K2048	A2512	91.93%	62.69%	98.93%	93.33%	91.72%
138542	6.00	C2129	D2303	Y2092	Y2131	A2512	W1677	91.93%	64.18%	98.57%	91.49%	92.00%
138549	6.00	C2129	D2303	Y2092	Y2131	K2048	A2512	91.93%	62.69%	98.93%	93.33%	91.72%
138828	6.00	C2129	D2303	A1410	Y2131	A2512	W1677	91.93%	64.18%	98.57%	91.49%	92.00%
