# Supplementary material for: Novel Genetic Variants of Hepatitis B Virus in Fulminant Hepatitis
Source: J Pathog. 2017 Dec 19;2017:1231204. doi: 10.1155/2017/1231204 (PMC5749291; doi:10.1155/2017/1231204)
Supplement: Supplementary 5 — Suppl Table 4: frequency of top 6 nucleotide variants in HBV isolates of fulminant and acute hepatitis. [file 1231204.f5.pdf]

SNP	C2129	T720	Y2131	T2013	K2048	A2512	Total number of variants
AB010290.1_genotype B_Japan_fulminant hepatitis	1	0	0	0	0	0	1
AB031265.1_genotype C_Vietnam_fulminant hepatitis	0	0	0	0	0	0	0
AB031266.1_genotype B_Vietnam_fulminant hepatitis	0	0	0	0	0	1	1
AB031267.1_genotype B_Vietnam_fulminant hepatitis	0	0	0	0	0	1	1
AB049609.1_genotype C_Japan_fulminant hepatitis	0	0	0	0	0	0	0
AB049610.1_genotype C_Japan_fulminant hepatitis	0	0	1	0	0	0	1
AB078031.2_genotype D_Japan_fulminant hepatitis	0	0	0	0	0	0	0
AB100695.1_genotype B_Vietnam_fulminant hepatitis	0	0	0	0	0	1	1
AB205152.1_genotype C_Japan_fulminant hepatitis	0	0	0	0	1	0	1
AB300368.1_genotype C_Japan_fulminant hepatitis	0	0	0	0	0	0	0
AB300369.1_genotype C_Japan_fulminant hepatitis	1	1	0	0	0	0	2
AB300370.1_genotype B_Japan_fulminant hepatitis	1	0	0	0	0	0	1
AB300371.1_genotype B_Japan_fulminant hepatitis	1	0	0	0	0	0	1
AB300372.1_genotype C_Japan_fulminant hepatitis	0	0	0	0	0	0	0
AB300373.1_genotype C_Japan_fulminant hepatitis	0	1	0	0	0	0	1
AB302942.1_genotype B_Japan_fulminant hepatitis	1	0	0	0	0	0	1
AB302943.1_genotype B_Japan_fulminant hepatitis	1	0	0	0	0	0	1
AB302944.1_genotype B_Japan_fulminant hepatitis	1	0	0	0	0	0	1
AB302945.1_genotype B_Japan_fulminant hepatitis	1	0	0	0	0	0	1
AB642091.1_genotype B_Japan_fulminant hepatitis	0	0	1	1	0	0	2
AB642092.1_genotype B_Japan_fulminant hepatitis	0	0	1	0	0	0	1
AB642093.1_genotype B_Japan_fulminant hepatitis	0	0	1	0	0	0	1
AB642094.1_genotype B_Japan_fulminant hepatitis	0	0	0	0	0	0	0
AB642095.1_genotype C_Japan_fulminant hepatitis	0	0	0	0	1	0	1
AB642096.1_genotype C_Japan_fulminant hepatitis	0	0	0	0	1	0	1
AB642097.1_genotype C_Japan_fulminant hepatitis	0	0	1	0	1	0	2
AB642098.1_genotype B_Japan_fulminant hepatitis	1	0	0	0	0	0	1
AB642099.1_genotype C_Japan_fulminant hepatitis	0	0	0	0	0	0	0
AB642100.1_genotype C_Japan_fulminant hepatitis	0	0	0	0	0	0	0
AF090838.1_genotype A_France_subfulminant hepatitis	0	0	0	1	0	0	1
AF297621.1_genotype A_South Africa_fulminant hepatitis	0	0	0	0	0	1	1
AF297622.1_genotype A_South Africa_fulminant hepatitis	1	0	0	0	0	0	1

SNP	C2129	T720	Y2131	T2013	K2048	A2512	Total number of variants
AF297623.1_genotype A_South Africa_fulminant hepatitis	0	0	1	0	0	0	1
AF297624.1_genotype A_South Africa_fulminant hepatitis	0	0	0	0	0	0	0
AF297625.1_genotype A_South Africa_fulminant hepatitis	0	0	0	0	0	0	0
AF458664.1_genotype C_China_fulminant hepatitis	0	0	0	0	0	0	0
AF458665.1_genotype C_China_fulminant hepatitis	0	0	0	0	0	1	1
AF461357.1_genotype C_China_fulminant hepatitis	0	0	0	0	0	0	0
AF461358.1_genotype C_China_fulminant hepatitis	0	0	0	0	0	0	0
AF461360.1_genotype B_China_fulminant hepatitis	0	0	0	0	0	0	0
AF461361.1_genotype C_China_fulminant hepatitis	0	0	0	0	0	0	0
AF461362.1_genotype B_China_fulminant hepatitis	0	0	0	0	0	0	0
AJ132335.1_genotype D_Italy_fulminant hepatitis	0	0	0	0	0	0	0
AJ627215.1_genotype D_Spain_fulminant hepatitis	0	0	0	1	0	0	1
AY066028.1_genotype C_China_fulminant hepatitis	0	0	0	0	0	0	0
AY233284.1_genotype A_South Africa_fulminant hepatitis	0	0	0	0	0	0	0
AY306136.1_genotype C_China_fulminant hepatitis	0	0	0	0	0	0	0
AY902768.1_genotype D_USA_fulminant hepatitis	0	1	0	0	0	0	1
AY902769.1_genotype D_USA_fulminant hepatitis	0	1	0	0	0	0	1
AY902770.1_genotype D_USA_fulminant hepatitis	0	1	0	0	0	0	1
AY902772.1_genotype D_USA_fulminant hepatitis	0	1	0	0	0	0	1
AY902774.1_genotype D_USA_fulminant hepatitis	0	1	0	0	0	0	1
AY902776.1_genotype D_USA_fulminant hepatitis	0	1	0	0	0	0	1
AY902777.1_genotype D_USA_fulminant hepatitis	0	1	0	0	0	0	1
D50521.1_genotype B_Japan_fulminant hepatitis	1	0	0	0	0	0	1
D50522.1_genotype B_Japan_fulminant hepatitis	1	0	0	0	0	0	1
GQ872211.1_genotype C_South Korea_fulminant hepatitis	0	0	0	0	0	0	0
L27106.1_genotype D_Israel_fulminant hepatitis	0	0	0	1	0	0	1
X80924.1_genotype D_UK_fulminant hepatitis	1	0	0	0	0	0	1
X97848.1_genotype D_UK_fulminant hepatitis	1	0	0	0	0	0	1
X97849.1_genotype D_UK_fulminant hepatitis	1	0	0	0	0	0	1
X97850.1_genotype B_UK_fulminant hepatitis	0	0	0	0	0	0	0
X97851.1_genotype B_UK_fulminant hepatitis	1	0	0	0	0	0	1
AJ627218.1_genotype D_Spain_fulminant hepatitis	0	0	0	1	0	0	1

SNP	C2129	T720	Y2131	T2013	K2048	A2512	Total number of variants
LC051141.1_genotype A_Japan_fulminant hepatitis	0	0	0	0	0	0	0
AB931168.1_genotype B_Japan_fulminant hepatitis	1	0	0	0	1	0	2
AB931170.1_genotype C_Japan_fulminant hepatitis	0	0	0	0	0	0	0
AB031262.1_genotype C_Vietnam_acute hepatitis	0	0	0	0	0	0	0
AB078032.1_genotype D_Japan_acute hepatitis	0	0	0	0	0	0	0
AB113879.1_genotype C_Japan_acute hepatitis	0	0	0	0	0	0	0
AB116266.1_genotype D_Japan_acute hepatitis	0	0	0	0	0	0	0
AB120308.1_genotype D_Japan_acute hepatitis	0	0	0	0	0	0	0
AB231908.1_genotype I_Vietnam_acute hepatitis	0	0	0	0	0	0	0
AB266536.1_genotype H_Japan_acute hepatitis	0	0	0	0	0	0	0
AB275308.1_genotype H_Japan_acute hepatitis	0	0	0	0	0	0	0
AB298720.1_genotype C_Japan_acute hepatitis	0	0	0	0	0	0	0
AB299858.1_genotype C_Japan_acute hepatitis	0	0	0	0	0	0	0
AB300359.1_genotype C_Japan_acute hepatitis	0	0	0	0	0	0	0
AB300360.1_genotype C_Japan_acute hepatitis	0	0	0	0	0	0	0
AB300361.1_genotype C_Japan_acute hepatitis	0	0	0	0	0	0	0
AB300362.1_genotype C_Japan_acute hepatitis	0	0	0	0	0	0	0
AB300363.1_genotype C_Japan_acute hepatitis	0	0	0	0	0	0	0
AB300364.1_genotype B_Japan_acute hepatitis	0	0	0	0	0	0	0
AB300365.1_genotype C_Japan_acute hepatitis	0	0	0	0	0	0	0
AB300366.1_genotype A_Japan_acute hepatitis	0	0	0	0	0	0	0
AB300367.1_genotype A_Japan_acute hepatitis	0	0	0	0	0	0	0
AB602818.1_genotype B_Japan_acute hepatitis	0	0	0	0	0	0	0
AY233274.1_genotype A_South Africa_acute hepatitis	0	0	0	0	0	0	0
AY233279.1_genotype A_South Africa_acute hepatitis	0	0	0	0	0	0	0
AY233283.1_genotype A_South Africa_acute hepatitis	0	0	0	0	0	0	0
AY233287.1_genotype A_South Africa_acute hepatitis	0	0	0	0	0	0	0
AY233292.1_genotype D_South Africa_acute hepatitis	0	0	0	0	0	0	0
AY902773.1_genotype D_USA_acute hepatitis	0	1	0	0	0	0	1
DQ991753.2_genotype D_Ireland_acute hepatitis	0	0	0	0	0	0	0
EF157291.1_genotype H_Japan_acute hepatitis	0	0	0	0	0	0	0
EU859898.1_genotype A_Belgium_acute hepatitis	0	0	0	0	0	0	0

SNP	C2129	T720	Y2131	T2013	K2048	A2512	Total number of variants
EU859899.1_genotype A_Belgium_acute hepatitis	0	0	0	0	0	0	0
EU859900.1_genotype A_Belgium_acute hepatitis	0	0	0	0	0	0	0
EU859901.1_genotype A_Belgium_acute hepatitis	0	0	0	0	0	0	0
EU859902.1_genotype A_Belgium_acute hepatitis	0	0	0	0	0	0	0
EU859903.1_genotype A_Belgium_acute hepatitis	0	0	0	0	0	0	0
EU859904.1_genotype A_Belgium_acute hepatitis	0	0	0	0	0	0	0
EU859905.1_genotype A_Belgium_acute hepatitis	0	0	0	0	0	0	0
EU859906.1_genotype A_Belgium_acute hepatitis	0	0	0	0	0	0	0
EU859907.1_genotype A_Belgium_acute hepatitis	0	0	0	0	0	0	0
EU859908.1_genotype A_Belgium_acute hepatitis	0	0	0	0	0	0	0
EU859909.1_genotype A_Belgium_acute hepatitis	0	0	0	0	0	0	0
EU859910.1_genotype A_Belgium_acute hepatitis	0	0	0	0	0	0	0
EU859911.1_genotype A_Belgium_acute hepatitis	0	0	0	0	0	0	0
EU859912.1_genotype A_Belgium_acute hepatitis	0	0	0	0	0	0	0
EU859913.1_genotype A_Belgium_acute hepatitis	0	0	0	0	0	0	0
EU859914.1_genotype A_Belgium_acute hepatitis	0	0	0	0	0	0	0
EU859915.1_genotype A_Belgium_acute hepatitis	0	0	0	0	0	0	0
EU859916.1_genotype A_Belgium_acute hepatitis	0	0	0	0	0	0	0
EU859917.1_genotype A_Belgium_acute hepatitis	0	0	0	0	0	0	0
EU859918.1_genotype A_Belgium_acute hepatitis	0	0	0	0	0	0	0
EU859919.1_genotype A_Belgium_acute hepatitis	0	0	0	0	0	0	0
EU859920.1_genotype A_Belgium_acute hepatitis	0	0	0	0	0	0	0
EU859921.1_genotype A_Belgium_acute hepatitis	0	0	0	0	0	0	0
EU859922.1_genotype A_Belgium_acute hepatitis	0	0	0	0	0	0	0
EU859923.1_genotype A_Belgium_acute hepatitis	0	0	0	0	0	0	0
EU859924.1_genotype A_Belgium_acute hepatitis	0	0	0	0	0	0	0
EU859925.1_genotype A_Belgium_acute hepatitis	0	0	0	0	0	0	0
EU859926.1_genotype A_Belgium_acute hepatitis	0	0	0	0	0	0	0
EU859927.1_genotype A_Belgium_acute hepatitis	0	0	0	0	0	0	0
EU859928.1_genotype A_Belgium_acute hepatitis	0	0	0	0	0	0	0
FJ349229.1_genotype D_Belgium_acute hepatitis	0	0	0	0	0	0	0
FJ349232.1_genotype D_Belgium_acute hepatitis	0	0	0	0	0	0	0

SNP	C2129	T720	Y2131	T2013	K2048	A2512	Total number of variants
GQ377514.1_genotype C_China_acute hepatitis	0	0	0	0	0	0	0
GQ377515.1_genotype C_China_acute hepatitis	0	0	0	0	0	0	0
GQ377516.1_genotype C_China_acute hepatitis	0	0	0	0	0	0	0
GQ377517.1_genotype C_China_acute hepatitis	0	0	0	0	0	0	0
GQ377518.1_genotype C_China_acute hepatitis	0	0	0	0	0	0	0
GQ377519.1_genotype B_China_acute hepatitis	0	0	0	0	0	0	0
GQ377520.1_genotype C_China_acute hepatitis	0	0	0	0	0	0	0
GQ377521.1_genotype C_China_acute hepatitis	0	0	0	0	0	0	0
GQ377522.1_genotype C_China_acute hepatitis	0	0	0	0	0	0	0
GQ377523.1_genotype C_China_acute hepatitis	0	0	0	0	0	0	0
GQ377524.1_genotype C_China_acute hepatitis	0	0	0	0	0	0	0
GQ377525.1_genotype B_China_acute hepatitis	0	0	0	0	0	0	0
GQ377526.1_genotype C_China_acute hepatitis	0	0	0	0	0	0	0
GQ377527.1_genotype C_China_acute hepatitis	0	0	0	0	0	0	0
GQ377528.1_genotype C_China_acute hepatitis	0	0	0	0	0	0	0
GQ377529.1_genotype C_China_acute hepatitis	0	0	0	0	0	0	0
GQ377530.1_genotype C_China_acute hepatitis	0	0	0	0	0	0	0
GQ377531.1_genotype C_China_acute hepatitis	0	0	0	0	0	0	0
GQ377532.1_genotype D_China_acute hepatitis	0	0	0	0	0	0	0
GQ377533.1_genotype C_China_acute hepatitis	0	0	0	0	0	0	0
GQ377534.1_genotype C_China_acute hepatitis	0	0	0	0	0	0	0
GQ377535.1_genotype C_China_acute hepatitis	0	0	0	0	0	0	0
GQ377536.1_genotype C_China_acute hepatitis	0	0	0	0	0	0	0
GQ377537.1_genotype B_China_acute hepatitis	0	0	0	0	0	0	0
GQ377538.1_genotype C_China_acute hepatitis	0	0	0	0	0	0	0
GQ377539.1_genotype C_China_acute hepatitis	0	0	0	0	0	0	0
GQ377540.1_genotype C_China_acute hepatitis	0	0	0	0	0	0	0
GQ377541.1_genotype C_China_acute hepatitis	0	0	0	0	0	0	0
GQ377542.1_genotype B_China_acute hepatitis	0	0	0	0	0	0	0
GQ377543.1_genotype C_China_acute hepatitis	0	0	0	0	0	0	0
GQ377544.1_genotype C_China_acute hepatitis	0	0	0	0	0	0	0
GQ377545.1_genotype C_China_acute hepatitis	0	0	0	0	0	0	0

SNP	C2129	T720	Y2131	T2013	K2048	A2512	Total number of variants
GQ377546.1_genotype C_China_acute hepatitis	0	0	0	0	0	0	0
GQ377547.1_genotype B_China_acute hepatitis	0	0	0	0	0	0	0
GQ377548.1_genotype C_China_acute hepatitis	0	0	0	0	0	0	0
GQ377549.1_genotype C_China_acute hepatitis	0	0	0	0	0	0	0
GQ377550.1_genotype B_China_acute hepatitis	0	0	0	0	0	0	0
GQ377551.1_genotype C_China_acute hepatitis	0	0	0	0	0	0	0
GQ377552.1_genotype C_China_acute hepatitis	0	0	0	0	0	0	0
GQ377553.1_genotype C_China_acute hepatitis	0	0	0	0	0	0	0
GQ377554.1_genotype C_China_acute hepatitis	0	0	0	0	0	0	0
GQ377555.1_genotype C_China_acute hepatitis	0	0	0	0	0	0	0
GQ377556.1_genotype C_China_acute hepatitis	0	0	0	0	0	0	0
GQ377557.1_genotype C_China_acute hepatitis	0	0	0	0	0	0	0
GQ377558.1_genotype B_China_acute hepatitis	0	0	0	0	0	0	0
GQ377559.1_genotype C_China_acute hepatitis	0	0	0	0	0	0	0
GQ377560.1_genotype C_China_acute hepatitis	0	0	0	0	0	0	0
GQ377561.1_genotype B_China_acute hepatitis	0	0	0	0	0	0	0
GQ377562.1_genotype C_China_acute hepatitis	0	0	0	0	0	0	0
GQ377563.1_genotype C_China_acute hepatitis	0	0	0	0	0	0	0
GQ377564.1_genotype C_China_acute hepatitis	0	0	0	0	0	0	0
GQ377565.1_genotype C_China_acute hepatitis	0	0	0	0	0	0	0
GQ377566.1_genotype B_China_acute hepatitis	0	0	0	0	0	0	0
GQ377567.1_genotype B_China_acute hepatitis	0	0	0	0	0	0	0
GQ377568.1_genotype B_China_acute hepatitis	0	0	0	0	0	0	0
GQ377569.1_genotype B_China_acute hepatitis	0	0	0	0	0	0	0
GQ377570.1_genotype C_China_acute hepatitis	0	0	0	0	0	0	0
GQ377571.1_genotype C_China_acute hepatitis	0	0	0	0	0	0	0
GQ377572.1_genotype C_China_acute hepatitis	0	0	0	0	0	0	0
GQ377573.1_genotype C_China_acute hepatitis	0	0	0	0	0	0	0
GQ377574.1_genotype C_China_acute hepatitis	0	0	0	0	0	0	0
GQ377575.1_genotype C_China_acute hepatitis	0	0	0	0	0	0	0
GQ377576.1_genotype C_China_acute hepatitis	0	0	0	0	0	0	0
GQ377577.1_genotype C_China_acute hepatitis	0	0	0	0	0	0	0

SNP	C2129	T720	Y2131	T2013	K2048	A2512	Total number of variants
GQ377578.1_genotype C_China_acute hepatitis	0	0	0	0	0	0	0
GQ377579.1_genotype C_China_acute hepatitis	0	0	0	0	0	0	0
GQ377580.1_genotype C_China_acute hepatitis	0	0	0	0	0	0	0
GQ377581.1_genotype C_China_acute hepatitis	0	0	0	0	0	0	0
GQ377582.1_genotype B_China_acute hepatitis	0	0	0	0	0	0	0
GQ377583.1_genotype C_China_acute hepatitis	0	0	0	0	0	0	0
GQ377584.1_genotype C_China_acute hepatitis	0	0	0	0	0	0	0
GQ377585.1_genotype C_China_acute hepatitis	0	0	0	0	0	0	0
GQ377586.1_genotype C_China_acute hepatitis	0	0	0	0	0	0	0
GQ377587.1_genotype B_China_acute hepatitis	0	0	0	0	0	0	0
GQ377588.1_genotype B_China_acute hepatitis	0	0	0	0	0	0	0
GQ377589.1_genotype D_China_acute hepatitis	0	0	0	0	0	0	0
GQ377590.1_genotype C_China_acute hepatitis	0	0	0	0	0	0	0
GQ377591.1_genotype C_China_acute hepatitis	0	0	0	0	0	0	0
GQ377592.1_genotype C_China_acute hepatitis	0	0	0	0	0	0	0
GQ377593.1_genotype C_China_acute hepatitis	0	0	0	0	0	0	0
GQ377594.1_genotype C_China_acute hepatitis	0	0	0	0	0	0	0
GQ377595.1_genotype B_China_acute hepatitis	0	0	0	0	0	0	0
GQ377596.1_genotype C_China_acute hepatitis	0	0	0	0	0	0	0
GQ377597.1_genotype C_China_acute hepatitis	0	0	0	0	0	0	0
GQ377598.1_genotype C_China_acute hepatitis	0	0	0	0	0	0	0
GQ377599.1_genotype C_China_acute hepatitis	0	0	0	0	0	0	0
GQ377600.1_genotype C_China_acute hepatitis	0	0	0	0	0	0	0
GQ377601.1_genotype C_China_acute hepatitis	0	0	0	0	0	0	0
GQ377602.1_genotype C_China_acute hepatitis	0	0	0	0	0	0	0
GQ377603.1_genotype C_China_acute hepatitis	0	0	0	0	0	0	0
GQ377604.1_genotype C_China_acute hepatitis	0	0	0	0	0	0	0
GQ377605.1_genotype C_China_acute hepatitis	0	0	0	0	0	0	0
GQ377606.1_genotype B_China_acute hepatitis	0	0	0	0	0	0	0
GQ377607.1_genotype C_China_acute hepatitis	0	0	0	0	0	0	0
GQ377608.1_genotype C_China_acute hepatitis	0	0	0	0	0	0	0
GQ377609.1_genotype C_China_acute hepatitis	0	0	0	0	0	0	0

SNP	C2129	T720	Y2131	T2013	K2048	A2512	Total number of variants
GQ377610.1_genotype B_China_acute hepatitis	0	0	0	0	0	0	0
GQ377611.1_genotype C_China_acute hepatitis	0	0	0	0	0	0	0
GQ377612.1_genotype B_China_acute hepatitis	0	0	0	0	0	0	0
GQ377613.1_genotype C_China_acute hepatitis	0	0	0	0	0	0	0
GQ377614.1_genotype C_China_acute hepatitis	0	0	0	0	0	0	0
GQ377615.1_genotype C_China_acute hepatitis	0	0	0	0	0	0	0
GQ377616.1_genotype C_China_acute hepatitis	0	0	0	0	0	0	0
GQ377617.1_genotype C_China_acute hepatitis	0	0	0	0	0	0	0
GQ377618.1_genotype C_China_acute hepatitis	0	0	0	0	0	0	0
GQ377619.1_genotype C_China_acute hepatitis	0	0	0	0	0	0	0
GQ377620.1_genotype C_China_acute hepatitis	0	0	0	0	0	0	0
GQ377621.1_genotype C_China_acute hepatitis	0	0	0	0	0	0	0
GQ377622.1_genotype B_China_acute hepatitis	0	0	0	0	0	0	0
GQ377623.1_genotype C_China_acute hepatitis	0	0	0	0	0	0	0
GQ377624.1_genotype C_China_acute hepatitis	0	0	0	0	0	0	0
GQ377625.1_genotype B_China_acute hepatitis	0	0	0	0	0	0	0
GQ377626.1_genotype C_China_acute hepatitis	0	0	0	0	0	0	0
GQ377627.1_genotype D_China_acute hepatitis	0	0	0	0	0	0	0
GQ377628.1_genotype C_China_acute hepatitis	0	0	0	0	0	0	0
GQ377629.1_genotype B_China_acute hepatitis	0	0	0	0	0	0	0
GQ377630.1_genotype C_China_acute hepatitis	0	0	0	0	0	0	0
GQ377631.1_genotype C_China_acute hepatitis	0	0	0	0	0	0	0
GQ377632.1_genotype C_China_acute hepatitis	0	0	0	0	0	0	0
GQ377633.1_genotype C_China_acute hepatitis	0	0	0	0	0	0	0
GQ377634.1_genotype C_China_acute hepatitis	0	0	0	0	0	0	0
GQ377635.1_genotype C_China_acute hepatitis	0	0	0	0	0	0	0
GQ377636.1_genotype C_China_acute hepatitis	0	0	0	0	0	0	0
GQ377637.1_genotype C_China_acute hepatitis	0	0	0	0	0	0	0
GQ377638.1_genotype B_China_acute hepatitis	0	0	0	0	0	0	0
GQ377639.1_genotype B_China_acute hepatitis	0	0	0	0	0	0	0
GQ377640.1_genotype C_China_acute hepatitis	0	0	0	0	0	0	0
GQ377641.1_genotype B_China_acute hepatitis	0	0	0	0	0	0	0

SNP	C2129	T720	Y2131	T2013	K2048	A2512	Total number of variants
GQ377642.1_genotype C_China_acute hepatitis	0	0	0	0	0	0	0
GQ377643.1_genotype B_China_acute hepatitis	0	0	0	0	0	0	0
GQ377644.1_genotype B_China_acute hepatitis	0	0	0	0	0	0	0
LC036263.1_genotype B_Japan_acute hepatitis	0	0	0	0	0	0	0
KR230749.1_genotype G_UK_acute hepatitis	0	0	0	0	0	0	0
AB937791.1_genotype A_Japan_acute hepatitis	0	0	0	0	0	0	0
AB937792.1_genotype A_Japan_acute hepatitis	0	0	0	0	0	0	0
AB937793.1_genotype A_Japan_acute hepatitis	0	0	0	0	0	0	0
AB937794.1_genotype A_Japan_acute hepatitis	0	0	0	0	0	0	0
AB971715.1_genotype C_Japan_acute hepatitis	0	0	0	0	0	0	0
KJ843163.1_genotype F_Argentina_acute hepatitis	0	0	0	0	0	0	0
KJ843164.1_genotype F_Argentina_acute hepatitis	0	0	0	0	0	0	0
KJ843165.1_genotype B_Argentina_acute hepatitis	0	0	0	0	0	0	0
KJ843166.1_genotype A_Argentina_acute hepatitis	0	0	0	0	0	0	0
KJ843167.1_genotype F_Argentina_acute hepatitis	0	0	0	0	0	0	0
KJ843168.1_genotype F_Argentina_acute hepatitis	0	0	0	0	0	0	0
KJ843169.1_genotype F_Argentina_acute hepatitis	0	0	0	0	0	0	0
KJ843170.1_genotype F_Argentina_acute hepatitis	0	0	0	0	0	0	0
KJ843171.1_genotype F_Argentina_acute hepatitis	0	0	0	0	0	0	0
KJ843172.1_genotype A_Argentina_acute hepatitis	1	0	0	0	0	0	1
KJ843173.1_genotype A_Argentina_acute hepatitis	0	0	0	0	0	0	0
KJ843174.1_genotype F_Argentina_acute hepatitis	0	0	0	0	0	0	0
KJ843175.1_genotype F_Argentina_acute hepatitis	0	0	0	0	0	0	0
KJ843176.1_genotype F_Argentina_acute hepatitis	0	0	0	0	0	0	0
KJ843177.1_genotype F_Argentina_acute hepatitis	0	0	0	0	0	0	0
KJ843178.1_genotype F_Argentina_acute hepatitis	0	0	0	0	0	0	0
KJ843179.1_genotype F_Argentina_acute hepatitis	0	0	0	0	0	0	0
KJ843180.1_genotype F_Argentina_acute hepatitis	0	0	0	0	0	0	0
KJ843181.1_genotype F_Argentina_acute hepatitis	0	0	0	0	0	0	0
KJ843182.1_genotype A_Argentina_acute hepatitis	0	0	0	0	0	0	0
KJ843183.1_genotype A_Argentina_acute hepatitis	0	0	0	0	0	0	0
KJ843184.1_genotype A_Argentina_acute hepatitis	0	0	0	0	0	0	0

SNP	C2129	T720	Y2131	T2013	K2048	A2512	Total number of variants
KJ843185.1_genotype F_Argentina_acute hepatitis	0	0	0	0	0	0	0
KJ843186.1_genotype A_Argentina_acute hepatitis	0	0	0	0	0	0	0
KJ843187.1_genotype D_Argentina_acute hepatitis	0	0	0	0	0	0	0
KJ843188.1_genotype A_Argentina_acute hepatitis	0	0	0	0	0	0	0
KJ843189.1_genotype F_Argentina_acute hepatitis	0	0	0	0	0	0	0
KJ843190.1_genotype F_Argentina_acute hepatitis	0	0	0	0	0	0	0
KJ843191.1_genotype F_Argentina_acute hepatitis	0	0	0	0	0	0	0
KJ843192.1_genotype A_Argentina_acute hepatitis	0	0	0	0	0	0	0
KJ843193.1_genotype F_Argentina_acute hepatitis	0	0	0	0	0	0	0
KJ843194.1_genotype F_Argentina_acute hepatitis	0	0	0	0	0	0	0
KJ843195.1_genotype F_Argentina_acute hepatitis	0	0	0	0	0	0	0
KJ843196.1_genotype F_Argentina_acute hepatitis	0	0	0	0	0	0	0
KJ843197.1_genotype F_Argentina_acute hepatitis	0	0	0	0	0	0	0
KJ843198.1_genotype F_Argentina_acute hepatitis	0	0	0	0	0	0	0
KJ843199.1_genotype F_Argentina_acute hepatitis	0	0	0	0	0	0	0
KJ843200.1_genotype F_Argentina_acute hepatitis	0	0	0	0	0	0	0
KJ843201.1_genotype F_Argentina_acute hepatitis	0	0	0	0	0	0	0
KJ843202.1_genotype F_Argentina_acute hepatitis	0	0	0	0	0	0	0
KJ843203.1_genotype F_Argentina_acute hepatitis	0	0	0	0	0	0	0
KJ843204.1_genotype F_Argentina_acute hepatitis	0	0	0	0	0	0	0
KJ843205.1_genotype F_Argentina_acute hepatitis	0	0	0	0	0	0	0
KJ843206.1_genotype F_Argentina_acute hepatitis	0	0	0	0	0	0	0
KJ843207.1_genotype F_Argentina_acute hepatitis	0	0	0	0	0	0	0
KJ843208.1_genotype F_Argentina_acute hepatitis	0	0	0	0	0	0	0
KJ843209.1_genotype F_Argentina_acute hepatitis	0	0	0	0	0	0	0
KJ843210.1_genotype F_Argentina_acute hepatitis	0	0	0	0	0	0	0
KJ843211.1_genotype F_Argentina_acute hepatitis	0	0	0	0	0	0	0
KJ843212.1_genotype F_Argentina_acute hepatitis	0	0	0	0	0	0	0
KJ843213.1_genotype F_Argentina_acute hepatitis	0	0	0	0	0	0	0
KJ843214.1_genotype A_Argentina_acute hepatitis	0	0	0	0	0	0	0
KJ843215.1_genotype A_Argentina_acute hepatitis	0	0	0	0	0	0	0
KJ843216.1_genotype A_Argentina_acute hepatitis	0	0	0	0	0	0	0

SNP	C2129	T720	Y2131	T2013	K2048	A2512	Total number of variants
KJ843217.1_genotype A_Argentina_acute hepatitis	0	0	0	0	0	0	0
KJ843218.1_genotype A_Argentina_acute hepatitis	0	0	0	0	0	0	0
AB846650.1_genotype H_Japan_acute hepatitis	0	0	0	0	0	0	0
AB775198.1_genotype A_Japan_acute hepatitis	0	0	0	0	0	0	0
AB775199.1_genotype A_Japan_acute hepatitis	0	0	0	0	0	0	0
AB775200.1_genotype A_Japan_acute hepatitis	0	0	0	0	0	0	0
AB775201.1_genotype A_Japan_acute hepatitis	0	0	0	0	0	0	0
AB778116.1_genotype A_Japan_acute hepatitis	0	0	0	0	0	0	0
KC836877.1_genotype A_Japan_acute hepatitis	0	0	0	0	0	0	0
KC836878.1_genotype A_Japan_acute hepatitis	0	0	0	0	0	0	0
KC836879.1_genotype A_Japan_acute hepatitis	0	0	0	0	0	0	0
KC836880.1_genotype A_Japan_acute hepatitis	0	0	0	0	0	0	0
KC836881.1_genotype A_Japan_acute hepatitis	0	0	0	0	0	0	0
JX898686.1_genotype D_Sweden_acute hepatitis	0	0	0	0	0	0	0
JX898687.1_genotype D_Sweden_acute hepatitis	0	0	0	0	0	0	0
JX898688.1_genotype D_Sweden_acute hepatitis	0	0	0	0	0	0	0
JX898689.1_genotype D_Sweden_acute hepatitis	0	0	0	0	0	0	0
JX898690.1_genotype D_Sweden_acute hepatitis	0	0	0	0	0	0	0
JX898691.1_genotype D_Sweden_acute hepatitis	0	0	0	0	0	0	0
JX898692.1_genotype D_Sweden_acute hepatitis	0	0	0	0	0	0	0
JX898693.1_genotype D_Sweden_acute hepatitis	0	0	0	0	0	0	0
JX898694.1_genotype D_Sweden_acute hepatitis	0	0	0	0	0	0	0
JX898695.1_genotype D_Sweden_acute hepatitis	0	0	0	0	0	0	0
JX898696.1_genotype D_Sweden_acute hepatitis	0	0	0	0	0	0	0
JX898697.1_genotype D_Sweden_acute hepatitis	0	0	0	0	0	0	0
JX898698.1_genotype D_Sweden_acute hepatitis	0	0	0	0	0	0	0
JX898699.1_genotype D_Sweden_acute hepatitis	0	0	0	0	0	0	0
